# Neuronal BAG3 attenuates tau hyperphosphorylation, synaptic dysfunction, and cognitive deficits induced by traumatic brain injury via the regulation of autophagy-lysosome pathway

**DOI:** 10.1007/s00401-024-02810-1

**Published:** 2024-10-11

**Authors:** Nicholas Sweeney, Tae Yeon Kim, Cody T. Morrison, Liangping Li, Diana Acosta, Jiawen Liang, Nithin V. Datla, Julie A. Fitzgerald, Haoran Huang, Xianglan Liu, Gregory Huang Tan, Min Wu, Kate Karelina, Chelsea E. Bray, Zachary M. Weil, Douglas W. Scharre, Geidy E. Serrano, Takashi Saito, Takaomi C. Saido, Thomas G. Beach, Olga N. Kokiko-Cochran, Jonathan P. Godbout, Gail V. W. Johnson, Hongjun Fu

**Affiliations:** 1 Department of Neuroscience, College of Medicine, Ohio State University, Columbus, OH USA; 2 Biomedical Sciences Graduate Program, College of Medicine, Ohio State University, Columbus, OH USA; 3 Medical Scientist Training Program, College of Medicine, Ohio State University, Columbus, OH USA; 4 Department of Neuroscience, Rockefeller Neuroscience Institute, West Virginia University, Morgantown, WV USA; 5 Department of Neurology, College of Medicine, Ohio State University, Columbus, OH USA; 6 Banner Sun Health Research Institute, Sun City, AZ USA; 7 RIKEN Center for Brain Science, Laboratory for Proteolytic Neuroscience, Saitama, 351-0198 Japan; 8 Department of Neurocognitive Science, Institute of Brain Science, Nagoya City University Graduate School of Medical Sciences, Nagoya, 467-8601 Japan; 9 Department of Anesthesiology, University of Rochester, Rochester, NY USA; 10 Chronic Brain Injury Program, The Ohio State University, 175 Pomerene Hall, 1760 Neil Ave, Columbus, OH USA

**Keywords:** Alzheimer’s disease, Traumatic brain injury, BAG3, Tau, Gliosis, Autophagy-lysosome pathway, Synaptic dysfunction, Memory

## Abstract

Growing evidence supports that early- or middle-life traumatic brain injury (TBI) is a risk factor for developing Alzheimer’s disease (AD) and AD-related dementia (ADRD). Nevertheless, the molecular mechanisms underlying TBI-induced AD-like pathology and cognitive deficits remain unclear. In this study, we found that a single TBI (induced by controlled cortical impact) reduced the expression of BCL2-associated athanogene 3 (BAG3) in neurons and oligodendrocytes, which is associated with decreased proteins related to the autophagy-lysosome pathway (ALP) and increased hyperphosphorylated tau (ptau) accumulation in excitatory neurons and oligodendrocytes, gliosis, synaptic dysfunction, and cognitive deficits in wild-type (WT) and human tau knock-in (hTKI) mice. These pathological changes were also found in human cases with a TBI history and exaggerated in human AD cases with TBI. The knockdown of BAG3 significantly inhibited autophagic flux, while overexpression of BAG3 significantly increased it in vitro. Specific overexpression of neuronal BAG3 in the hippocampus attenuated AD-like pathology and cognitive deficits induced by TBI in hTKI mice, which is associated with increased ALP-related proteins. Our data suggest that targeting neuronal BAG3 may be a therapeutic strategy for preventing or reducing AD-like pathology and cognitive deficits induced by TBI.

**Supplementary Information:**

The online version contains supplementary material available at 10.1007/s00401-024-02810-1.

## Introduction

Traumatic brain injury (TBI) is a major cause of death and disability in the United States. Each day, TBI comprises about 30% of all injury-related deaths in the United States [95]. Military personnel and other individuals living with TBI face an increased risk of developing several long-term health problems including Alzheimer’s disease (AD) and AD-related dementias (ADRD) [7, 76]. AD is the most common form of dementia in the elderly with an estimated 6.7 million Americans aged 65 and older living with Alzheimer’s dementia in 2023. It is the seventh-leading cause of death in the United States (Alzheimer’s Association). Growing evidence supports that early, middle, and late life of TBI is a risk factor for developing late-life AD and ADRD [56, 68, 87, 111] although some studies challenge this association [18, 26, 35, 76, 103]. Moderate and severe TBIs and even mild TBI (mTBI) without loss of consciousness are found to be associated with an increased risk of dementia [21, 72, 76]. However, the molecular mechanisms underlying the TBI-induced AD-like pathology (e.g., amyloid accumulation, tau aggregation, synaptic dysfunction, gliosis, neuronal loss, etc.) and the cognitive deficits remain unknown.

Tau pathology, a major pathological hallmark in patients with AD [48], has also been detected in several TBI mouse models [23, 33, 37, 38, 45, 78, 91, 96, 107, 113] and human TBI patients as well as patients with the history of TBI [65, 68, 111]. TBI not only accelerates tau pathology in transgenic mice overexpressing mutant human APP, mutant human tau, or wild-type human tau [23, 74, 91, 97, 107, 112], but also can induce tau pathology in wild-type mice [23, 27, 44, 45, 66, 91, 107] and rats [25, 33]. Furthermore, experimental TBI can induce a self-propagation of tau pathology, which can be transmitted between mice [114, 116]. Despite many animal models of TBI showing increased tau pathology, several studies do not show significant changes in tau pathology or hyperphosphorylation after TBI [16, 47, 73]. The variability of TBI-induced tau pathology in mouse models may depend on the method of injury (i.e., fluid percussion injury, blast, controlled cortical impact, or closed head injury model of engineered rotational acceleration (CHIMERA)), the type of animal model used, the time post injury, or the type of tau antibody used. Thus, investigating whether TBI induces tau pathology in animal models is still important to understand mechanisms that drive TBI-induced neurodegeneration. Interestingly, tau pathology is closely related to other AD-like pathologies, such as synaptic dysfunction, gliosis, and neuronal loss [40, 41, 57]. Thus, tau pathology may serve as a causative link between TBI and AD as well as ADRD [51]. However, the molecular determinants responsible for TBI-induced tau pathology and neurodegeneration remain unclear.

We have recently identified BCL2-associated athanogene 3 (BAG3), which facilitates macroautophagy and thus likely attenuates protein aggregation [12, 42, 49, 52–54, 90], as a hub gene regulating tau homeostasis via single nucleus RNA-seq analysis of human postmortem brains from control donors [22]. We further verified that reducing BAG3 levels in primary neurons exacerbated pathological tau accumulation, whereas its overexpression attenuated tau accumulation [22], which is consistent with previous reports that BAG3 regulates tau clearance [12, 42, 49, 52–54, 90], and its level is reduced in excitatory (EX) neurons in human AD. At the same time, we noticed the immunoreactivity of BAG3 was increased in astrocytes in human AD [22], which might contribute to the absence of tau pathology in this cell type. This hypothesis has been validated by a recent study showing astrocyte BAG3 can protect against tau and alpha-synuclein pathologies and is upregulated in disease-associated astrocytes in human AD [90]. These results demonstrate that BAG3 determines the cellular vulnerability to tau pathology in human AD.

In this study, we aim to investigate the cell type-specific role of BAG3 in TBI-mediated pathology, such as tau hyperphosphorylation, gliosis, synaptic dysfunction, and memory loss, using both wild-type (WT) and humanized tau knock-in (hTKI) [31, 83] mouse models and human postmortem brains with or without TBI history. Interestingly, we found that a single TBI can reduce the level of BAG3 in EX neurons and oligodendrocytes (OLG) with ptau accumulation, while BAG3 level is increased in GFAP + astrocytes. Disruption of BAG3 homeostasis is associated with tau hyperphosphorylation in EX and OLG, gliosis, synaptic dysfunction, and memory loss in both WT and hTKI mice. Similar pathological changes were also found in human cases with a TBI history and further increased in human AD cases with TBI. The knockdown of BAG3 significantly inhibited autophagic flux, while overexpression of BAG3 significantly increased it in vitro. Importantly, specific overexpression of BAG3 in neurons attenuated AD-like pathology and cognitive deficits induced by TBI probably via the up-regulation of autophagy-lysosome pathway (ALP). Our findings demonstrate a cell type-specific role of BAG3 in TBI-mediated ALP dysfunction, AD-like pathology, and cognitive deficits (Supplementary Fig. 1).

## Methods

### Animals

Wild-type (WT) C57BL6/J mice were purchased from the Jackson Laboratory (Strain #: 000664). Human MAPT KI (hTKI) mice on a C57BL6/J background were generated by inserting the human *MAPT* gene at the murine *Mapt* gene locus [31, 83]. hTKI mice were provided by the RIKEN BioResource Research Center. All mice were maintained in a controlled environment (12-h light/dark cycle) with ad libitum access to food and water. Animal experiments were performed according to the approved animal protocols and national guidelines (National Institutes of Health), by the Institutional Animal Care and Use Committee (IACUC) of the Ohio State University. Mice were anesthetized by intraperitoneal administration of 100 mg/kg of ketamine (Covetrus North America, NDC Code: 11695-0703-1) and 10 mg/kg of xylazine (Akorn Animal Health, NDC Code: 59399-110-20), and transcardially perfused with 0.05% heparin in 1 × phosphate-buffered saline (PBS). Collected brains were either stored at −80 °C for total protein extraction or fixed in 10% formalin overnight at 4 °C for immunofluorescence (IF) staining. Formalin-fixed brains were cut into 35-μm-thick coronal sections using a cryostat microtome (CM1950, Leica Microsystems) and were stored at 4 °C in 1 × PBS with 0.02% sodium azide.

### Human postmortem brain tissues

Human fresh frozen brain blocks (inferior parietal lobe, IPL) were provided by the Arizona Study of Aging and Neurodegenerative Disorders/Brain and Body Donation Program at Banner Sun Health Research Institute [9]. A cryostat microtome (CM1950, Leica Microsystems) was used to cut fresh frozen brain blocks into 10-μm-thick sections for IF staining. Human brain tissues used in this study are listed in Table 1, including neuropathology and demographics of human cases. The focus of this study is the investigation between the relationship of tau and BAG3 and its potential implications in TBI pathophysiology. Given that the IPL is one of the brain regions often affected by TBI [11, 65, 80], this region was selected for analysis of human TBI cases. Unfortunately, there was no record of the exact brain region with TBI in the cases collected by Banner Sun Health Research Institute Brain and Body Donation Program, likely a result of the self-recall reporting of injury mentioned in the previous comment. Furthermore, the Banner Sun Health Research Institute Brain and Body Donation Program has a relatively small cohort of post-mortem TBI cases, which happen to have higher tau pathology than controls, but lower pathology than confirmed AD cases. Thus, control cases were selected with the lowest NIA-AA (i.e., A = 0, B = 1, C = 0) and TBI cases (without a confirmed AD diagnosis) were selected from all the available cases with TBI history with middle to high NIA-AA (i.e., A = 0–3, B = 2–3, C = 0–3) in Banner Health Institute’s Brain Bank. The AD cases with or without TBI were selected from cases with a confirmed AD diagnosis and high NIA-AA scores (i.e., A = 3, B = 3, C = 3).

**Table 1:** Post-mortem case demographics

Case ID	Sex	Age (yr)	APOE	TBI	TBI history	Last known MMSE	Amyloid thal phase (A)	Braak NFT stage (B)	CERAD neuritic plaque score (C)	Neuro-pathological diagnosis
CT-1	M	93	3/3	No	N/A	30	0	1	0	CWMR
CT-2	M	79	3/3	No	N/A	29	0	1	0	CWMR
CT-3	M	74	2/3	No	N/A	28	0	1	0	None
CT-4	F	75	3/3	No	N/A	N/A	0	1	0	None
CT-5	M	86	3/3	No	N/A	28	0	1	0	None
TBI-1	M	82	3/3	Yes	1st. possible LOC as child 2nd. several day LOC—plane crash 36 years from TBI to death	28	0	2	0	CWMR
TBI-2	F	77	3/4	Yes	1st. w/o LOC 3 months from TBI to death	27	3	2	3	ARG, CAA, MCI
TBI-3	F	93	3/3	Yes	1st. LOC —Fall; Occipital scalp hematoma days from TBI to death	30	3	3	3	TDP-43, CWMR, LBS
TBI-4	M	97	3/3	Yes	1st. w/o LOC—Fall; Fracture of left occipital bone 2nd. LOC—head injury; left subdural hematoma evacuation 3 months from TBI to death	27	1	2	1	MCI
TBI-5	F	90	3/3	Yes	1st. LOC—fall 22 months from TBI to death	25	3	2	3	ARG, TDP-43, CWMR, MCI
AD-1	M	82	2/4	No	N/A	21	3	3	3	ARG, CAA, AD
AD-2	F	84	3/3	No	N/A	28	3	3	3	MSA, ARG, AD
AD-3	F	86	3/3	No	N/A	23	3	3	3	CWMR, CAA, AD
AD-4	M	77	3/4	No	N/A	N/A	3	3	3	CWMR, CAA, AD
AD-5	M	76	3/3	No	N/A	12	3	3	3	CWMR, CAA, LBS, AD
ADwTBI-1	F	92	3/4	Yes	1st. 3 y/o, LOC; 2nd. 61 y/o, LOC 89 and 31 years from TBI to death, respectively	1	3	3	3	CAA, AD
ADwTBI-2	M	83	3/3	Yes	1st. LOC —Fall; Facial Trauma 2 years from TBI to death	18	3	3	3	PSP, CWMR, CAA, AD
ADwTBI-3	M	90	3/3	Yes	1st. LOC —fall; scalp laceration 1 month from TBI to death	24	3	3	3	CWMR, CAA, AD
ADwTBI-4	M	88	3/4	Yes	1st. LOC—Fall 2 years from TBI to death	18	3	3	3	HS, TDP-43, CWMR, CAA, AD

Human fresh frozen brain samples from the inferior parietal lobe (IPL) were used for both IF staining and Western Blot assay; All samples are classified according to the ABC scoring method described in the National Institute of Aging-Alzheimer’s Association guidelines for the neuropathologic assessment of AD (NIA-AA AD) [69]

*MMSE* mini-mental state examination, *AD* alzheimer’s disease, *TBI* traumatic brain injury, *LOC* loss of consciousness, *NDs* neurodegenerative diseases including TDP-43, progressive supranuclear palsy (PSP), hippocampal sclerosis (HS), multiple system atrophy (MSA), argyrophilic grain disease (ARG), cerebral white matter rarefaction (CWMR), mild cognitive impairment (MCI), lewy bodies (LBS), and cerebral amyloid angiopathy (CAA), *M* male, *F* female, *N/A* not available

### Reagents

Information for specific antibodies used in this study is listed in Table 2. Alexa Fluor dye-labeled cross-absorbed donkey secondary antibodies were purchased from ThermoFisher Scientific. TrueBlack Lipofuscin Autofluorescence Quencher (Cat# 23007) was purchased from Biotium. Sudan Black B (Cat# 199664), Hoechst33342 (Cat# 14533), and Heparin (Cat# 1003535076) were purchased from Sigma-Aldrich. Fluoromount-G Mounting Medium (Cat# 0100-01) was purchased from SouthernBiotech. Secondary antibodies for WB used were HRP-conjugated anti-mouse or anti-rabbit IgG antibodies and IRDye goat anti-rabbit or anti-mouse IgG secondary antibodies, purchased from LI-COR. Immobilon Western Chemiluminescent HRP substrate (WBKLS0500) was purchased from Millipore Sigma. Adeno-associated viruses (AAVs) (AAV9-hSYN1-eGFP-2A-hBAG3-WPRE and control AAV9-hSYN1-eGFP-2A-WPRE) and lentivirus (FUW mCherry-GFP-LC3) used in this study were provided by Vector Biolabs and Addgene, respectively. BAG3-related lentiviruses (i.e., FG12-scramble, FG12-shBAG3, shRNA-resistant BAG3, and shBAG3-BFP) were generated by Gail Johnson as previously described [22, 49, 53].

**Table 2:** Antibodies used for IF staining and WB assay in this study

Antibody	Source	Catalog number
Mouse anti-PHF1 (pSer396/pSer404tau)	Peter Davies	N/A
Mouse anti-CP13 (pSer202 tau)	Bioss	#bs-1124012-A647
Mouse anti-AT8 (pSer202/Thr205 tau)	ThermoFischer Scientific	#MN1020
Goat anti-Olig2	R&D systems	#AF2418
Mouse anti-PSD95	BioLegend	#810301
Chicken anti-GFAP	BioLegend	#829401
Mouse anti-SQSTM1(P62)	Abnova	#H00008878-M01
Rabbit anti-TauC	Sigma Aldrich	#A0024
Rabbit anti-BAG3	Proteintech	#10599-1-AP
Rabbit anti-CTSD	Proteintech	#21327-1-AP
Rabbit anti-TFEB	Proteintech	#13372-1-AP
Rabbit anti-SATB2	Abcam	#ab92446
Mouse anti-SATB2	Abcam	#ab51502
Rat anti-LAMP1	DSHB	#1D4B-C
Rabbit anti-IBA-1	FujiFilm Wako	#019-19741
Rabbit anti-GAPDH	Proteintech	#60004-1-Ig
Mouse anti-GAPDH	Proteintech	#10494-1-AP
Goat anti-GAD1	R&D systems	#AF2086
Goat anti-PDGFR-alpha	R&D systems	#AF1062
Mouse anti-SNAP-25	Abcam	#AB119028
Mouse anti-CC1	Calbiochem	#OP80-100UG
Rabbit anti-S396	Abcam	#AB109390

### Immunofluorescence (IF) staining

IF staining was performed as described previously [14, 22]. Free-floating sections from mouse brains were incubated with 10 mM sodium citrate antigen retrieval buffer (pH 6.0) at 95 °C for 12 min and were cooled to room temperature in antigen retrieval buffer for an additional 15 min. Following antigen retrieval, the floating sections were washed with 1 × PBS (3 times) and blocked with 10% donkey serum in 0.3% Triton X-100 (PBSTx) or 0.1% Tween20 (PBST20) for 1 h at room temperature. Then, the sections were incubated with primary antibodies at 4 °C overnight. After primary antibody incubation, sections were washed three times with either 0.1% PBSTx or 0.1% PBST20 (dependent on blocking buffer solution) and incubated with secondary antibodies (1:1000) diluted in blocking buffer at room temperature for 2 h. After three washes with 1 × PBS, floating sections were mounted, and 0.3% Sudan Black B in 70% ethanol was used to quench autofluorescence. Nuclei were visualized by 5 ug/mL Hoechst33342 in 0.3% PBST for 9 min at room temperature. Sections were sealed with Fluoromount-G Mounting Medium and were imaged using a Zeiss Axio Observer Microscope for epifluorescence images or a Leica SP8 imaging system for confocal images.

Some modifications to the above protocol were made to optimize for fresh frozen human sections. Slides were air-dried at 37 °C for 10 min and then fixed by 10% Formalin for 30 min (or overnight for microglia visualization). Then, the slides were washed by 1 × PBS and permeabilized by prechilled acetone at −20 °C for 15 min. After washing by 1 × PBS, slides were incubated in antigen retrieval buffer (as described above), washed by 1 × PBS, and blocked by 10% donkey serum in 0.3% PBSTx or 0.1% PBST20. Slides were then incubated at 4 °C overnight with primary antibodies. On the following day, slides were washed with 0.1% PBSTx or 0.1% PBST20 and incubated in corresponding secondary antibodies (1:1000) at room temperature for 2 h. Nuclei were visualized as described above. Autofluorescence was further quenched with 0.5% TrueBlack solution in 70% ethanol for 10 min, followed by three 1 × PBS washes. Sections were sealed and imaged in the same way as free-floating sections.

### Light-emitting diode (LED) array for quenching autofluorescence

Human fresh frozen sections contain autofluorescence, namely lipofuscin, that can confound the results of IF quantification. To reduce autofluorescence, we performed quenching via LED array as previously described [19], with some modifications. Human fresh frozen sections were air-dried at 37 °C and fixed with 10% formalin for 30 min (or overnight for microglia visualization). After three 1 × PBS washes, the slides were submerged in 1 × PBS and subjected to broad-spectrum LED light (7.5 cm distance from section) overnight in 4 °C refrigerator (at least 10 h). The slides were then visualized using a Zeiss Axio Observer Microscope to confirm the quenching of autofluorescence. Then, IF staining was performed as described earlier.

### Western blot (WB) assay

The WB assay was conducted to analyze the total protein fractions of fresh frozen human brains and snap-frozen mouse brains as previously described [14]*.* Prior to WB assay, the extraction of total protein was performed. 1.4-mm ceramic beads (Cat# 19-627-3, Cole-Parmer) were used to homogenize brain samples in 1 × RIPA buffer (Cat# 89901, ThermoFisher Scientific) with Protease and Phosphatase inhibitor cocktail (Cat# 78441, ThermoFisher Scientific) and 1 mM phenylmethyl-sulfonyl fluoride (PMSF, Cat# P7626, Sigma-Aldrich). Samples were centrifuged (5000 g, 20 min, 4 °C) and lysate was removed from the ceramic beads. Ultrasonication was performed to ensure complete homogenization of lysate. Protein concentrations were measured by Bicinchoninic Acid (BCA) Protein Assay Kits (Cat# 23225, ThermoFisher Scientific).

Total protein fractions were separated by electrophoresis by running 20 μg protein lysate on 4–12% Bis–Tris precast polyacrylamide gels and blotted using nitrocellulose blotting membranes (Cat# 10600001, Amersham Biosciences). The membranes were blocked using 5% milk in 0.1% TBS Tween20 for 1 h at room temperature and incubated with primary antibodies overnight at 4 °C on a shaker. After probing target proteins with primary antibodies, membranes were incubated with horseradish peroxidase (HRP)-conjugated or IRDye secondary antibodies at room temperature for 1.5 h. Membranes were imaged using a Li-Cor Odyssey Imager. HRP-conjugated blots were developed with enhanced chemiluminescence (ECL) WB detection reagents before imaging with Li-Cor Imager. Each protein band was measured using the Image Studio Lite software with glyceraldehyde 3-phosphate dehydrogenase (GAPDH), which was used as a loading control to normalize the levels of proteins loaded in the same gel.

### Controlled cortical impact (CCI) surgery

The CCI TBI procedure used in this study was performed as previously described [107] with minor modifications. Prior to Sham or CCI TBI, mice (10–12 mo.) were anesthetized in a closed plastic box connected to an Isoflurane system (Patterson Veterinary, CO, USA). Mice were then placed on a stereotaxic frame (RWD Life Science, China) with 2–3% isoflurane and 1–2% O_2_. After a midline skin incision, a 3.5-mm circular craniotomy was performed in the center of lambda and bregma, 1.0 mm to the right of the central suture using an electric drill. Prior to injury in TBI mice, the impactor was angled to make the tip of the impactor perpendicular to the exposed cortical surface. Then CCI was inflicted using an electric impact device (myNeuroLab, St. Louis, MO) with a 2.0 mm diameter flat impact tip (velocity = 3.00 m/sec, depth = 0.8 mm, and contact time = 200 ms). Cotton-tipped applicators were used to remove any blood following the injury without touching the injury area. The skin was sutured once the bleeding stopped. A heating pad (RWD Life Science, China) was used before and during the surgery to minimize heat loss. For injury assessment after wound closure, the animal is then placed on a heating pad, and the time to self-right is recorded as an indicator of the severity of the brain injury. Self-righting typically takes 1–5 min, while sham animals right themselves immediately, and those with a more severe TBI take as long as 8 min. The entire procedure, including injury assessment, lasts ~ 10–15 min or less. One month following Sham or TBI surgery, mice were subjected to a variety of behavior tests (see Methods). To avoid any effect of behavioral testing, mice were sacrificed 2 weeks later for IF analysis.

### Stereotaxic surgery

Stereotaxic viral injections were performed as previously described [14] in line with the IACUC guidelines of The Ohio State University. Mice (7–9 mo.) received anesthesia by placing them in a closed plastic box connected to an Isoflurane system (Patterson Veterinary, CO, USA) prior to surgery. Mice then were placed on a stereotaxic instrument (RWD Life Science, China) and maintained anesthesia via nose cone, allowing for constant flow of isoflurane (1.5–2% by volume) and 1–2% O_2_ throughout the surgery. Viruses were injected using a 10-μL Hamilton microsyringe (GASTIGHT #1701) attached with a 30-gage needle. A microsyringe pump (KD Scientific, MA, USA) was used to control the speed of injection at 100 nL/min. The delivery of AAV9-hSyn-eGFP-2A-hBAG3-WPRE or control AAV9-hSyn-eGFP-2A-WPRE viruses was directed into both hippocampal CA1 (500 nL, Anterior–posterior (AP): −1.95 mm, Medial–lateral (ML): -1.45 mm, Dorsal–ventral (DV): -2.05 mm, relative to bregma) and dentate gyrus (DG) (500 nL, AP: −1.95 mm, ML: −1.45 mm, DV: −1.6 mm, relative to bregma), according to the mouse brain atlas of Paxinos and Franklin’s (fourth edition). After injection, the syringe was left in place for an additional 10 min to minimize the reflux along the injection track. Mice were kept on a warming pad until they fully recovered from anesthesia and housed individually to prevent the damage of scalp sutures. After 3 months, CCI surgery (see Methods) was performed on AAV9-GFP and AAV9-BAG3-injected mice.

### Behavioral tests

#### Y-maze

The spatial working memory was evaluated by the Y-maze test after a month of CCI surgery, as previously described [14]. A Y-maze, constructed of three identical arms of opaque plastic (40 × 4.5 × 12 cm) 120° apart, was placed in the center of the room with a dim light of 30 lx brightness. Visual cues to allow for visual orientation were located on the wall of each arm. Before the experiment, mice were freely exposed to three arms (labeled “A”, “B”, “C”) of the maze for 10 min of the habituation phase. Each mouse was placed at the end of one arm facing the center of the Y-maze. The movement of each mouse was recorded with an overhead camera for 10 min and the entries to each arm were scored using an automatic video tracking system (ANY-maze). Spontaneous alternation performance was determined as successive entries of three different arms consecutively without repetition. The spontaneous alternation percentage was calculated as [(number of alternations) / (total arm entries − 2)] × 100. Mice with fewer than 2 total entries were excluded from the analysis. The maze was sterilized with 70% ethyl alcohol, between each test, to eliminate any scent left behind by previously tested mice.

#### Open-field test

Open-field test assesses the alteration of the exploratory and locomotor activity. The open-field test was performed after a month of CCI surgery in both WT and hTKI mice and evaluated using the Basso Mouse Scale [8]. Each mouse was placed at the same side of a circular plastic platform (12″ × 12″ × 12″), where animals are allowed to explore the enclosed space freely for 4 min. Each trail was recorded, and the time of resting was measured. The chamber was sterilized with 70% ethyl alcohol between each trial and the testing room conditions were controlled equally.

#### Morris water maze

Spatial learning and memory were evaluated using the Morris water maze test including hidden platform acquisition and probe trial test. Each mouse was tested in a cylindrical tank filled with opaque water (25 °C). The tank was divided into 4 equal quadrants with a transparent platform 1.5 cm below the water surface. Each quadrant involved different navigation landmarks and all tests were recorded by ANY-maze video tracking software. During the hidden platform acquisition test, each mouse was placed at each quadrant/trial, allowing them to swim freely and search for the escape platform within 60 s. The escape latency, the time and the distance taken to reach the escape platform, was measured. Mice that failed to find the escape platform within 60 s were recorded as 60 s. After each trial, the mouse was kept on the platform for 10 s and the next trial from a different quadrant was conducted after 4 min. The experiment was repeated for 4 consecutive days and the mean of the escape latency was measured. The probe trial test was conducted by removing the platform, 2 and 24 h after the hidden platform acquisition test. Each mouse was placed in the diagonal quadrant of the platform that was located before and was allowed to swim freely for 60 s. The time and the distance spent in the target (previous hidden platform) were measured to quantify the short- and the long-term memory maintenance.

#### Measurement of autophagy in vitro in HEK293 cells

HEK293 cells were seeded at a density of 1 × 10^5^/mL on coverslips coated with Poly-L-Ornithine (PLO, 15 μg/mL). Twenty-four hours after seeding, cells were treated with a scr-BAG3, sh-BAG3-BFP, sh-BAG3, or sh-res-BAG3 lentiviruses. Twenty-four hours after lentiviral transduction, cells were then treated with the FUW-GFP-mCherry-LC3 autophagy reporter virus. Forty-eight hours after treatment with the FUW-GFP-mCherry-LC3 reporter, cells were live-imaged using a Zeiss Axio Observer Microscope and assessed for the formation of single red puncta. After imaging, cells were treated with Bafilomaycin-A1 (BafA1; Cat # B1793) for 6 h and live-imaged again to assess formation of yellow puncta. Puncta were manually counted using Zeiss Zen microscope software (2.6 blue edition). Statistical analysis and plots were generated using GraphPad Prism (version 9.5.1).

#### Analysis of immunofluorescence staining

For mouse brain IF experiments, tile images were taken at the same exposure times between groups. To complete PHF1 + or CP13 + cell counting, automated cell counting in ImageJ (NIH) was used. Briefly, images were exported as TIFF files and converted to 8-bit. Then regions of interest (ROI) were selected according to Supplementary Fig. 3. Then, the Find Edges and Sharpen functions were used prior to thresholding. The images were then watershed, and the analyzed particle function was used to count the number of positive cells. To normalize the number of PHF1 or CP13 + cells based on the area quantified, the set scale function was used in ImageJ. For mean intensity or percentage of area measurements (i.e., GFAP, IBA-1, PSD95, SNAP25, CTSD, LAMP1, and BAG3), tile images were exported as TIFF files at the same parameters. The images were added to ImageJ, then converted to 8-bit, and the measure function was used to determine the mean intensity or the percentage of area. ROIs were selected according to Supplementary Fig. 3j. For mean intensity measurements of BAG3 in GFAP + cells, the CA1 was selected due to the large number of astrocytes in both groups, well-defined morphology, and proximity to the injury site. Cells were manually defined using the Draw Spline Contour in Zeiss Zen (2.6 blue edition) software. Mean intensity values were given by the software after annotation. For the mean intensity of BAG3 in PHF1 + or PHF1- cells, PHF1 + or PHF1- neurons and oligodendrocytes were manually annotated in the cortex zones proximal to the injury site. Again, cells were manually defined using the Draw Spline Contour in Zeiss Zen (2.6 blue edition). Mean intensity values were given by the software after annotation. The number of GFAP + cells or PHF1 + /PHF1- cells was selected based on our previous publication [22]. The number of cells with aggregated P62 puncta was quantified in the cortical region proximal to the injury site. Based on interest of cells with accumulated P62 puncta, positive cells were manually annotated using Zeiss Zen (2.6 blue edition). The contour tool in Zeiss Zen (2.6 blue edition) was used to obtain the area that the number of P62-positive cells was counted within.

For human brain IF experiments, tiles images were taken similar to the mouse IF quantification. For mean intensity-based measurements (i.e., GFAP, IBA-1, AT8, PHF1, BAG3, CTSD, LAMP1, P62, and BAG3), ImageJ was used to measure the mean intensity similar to the mouse IF quantification. Based on ptau prevalence in the gray matter, quantification included all cortical layers but not white matter regions. Measurement of BAG3 in GFAP + cells and PHF1 + /PHF1- cells followed the methods described in the mouse brain analyses. For the analysis of the number of IBA1 + and GFAP + cells close to and within to neuritic plaques, the number of IBA1 + and GFAP + cells was manually counted in Zeiss Zen (2.6 blue edition) with an ROI of 430 μm by 420 µm including the plaque. The same size ROI was also used for control cases that lacked tau pathology. Further, the majority of neuritic plaques analyzed could be found in layers V and VI. Thus, ROIs for control cases were also selected from layers V and VI of the IPL. Neuritic plaques were defined as extracellular tau aggregates that lacked a central nucleus while PHF1 + cells were defined as cells containing PHF1 with a clear nucleus as described previously [34]. To quantitate the number of PHF1 + neuritic plaques or the number of PHF1 + cells, the number of each was manually counted using Zeiss Zen (2.6 blue edition). To normalize the number of plaques or cells to the area quantified, the spline contour tool was used in Zeiss Zen (2.6 blue edition) to obtain the area quantified.

#### Statistical analysis

Sample sizes were not predetermined using any statistical methods but followed sample sizes pre-established in the literature. Prism 9 software was used to analyze data. All data are expressed as mean ± SEM. For the normality test, we utilized the Shapiro–Wilk test and Kolmogorov–Smirnov test based on the relatively small sample size of our data. Based on the results of the normality test, we used nonparametric Mann–Whitney test or unpaired *t*-test to compare the mean intensity of IF staining (PHF1, AT8, GFAP, IBA-1, BAG3, PSD95, SNAP-25, CTSD, LAMP1, and p62) or the numbers of marker-positive cells (PHF1, CP13, and P62). All results represent two-sided tests comparing biological replicates between groups. *P* < 0.05 was considered statistically significant for all measures. The n values represent the number of mice, cases, cells, or images in each group that were quantified. The n values are indicated in the figure legends for easy association with their respective tests. See figure legends for the detailed statistical information that corresponds with each experiment.

## Results

### TBI increases tau hyperphosphorylation, gliosis, synaptic dysfunction, and memory deficits in both wild-type (WT) and humanized tau knock-in (hTKI) mice

**Fig. 1:**
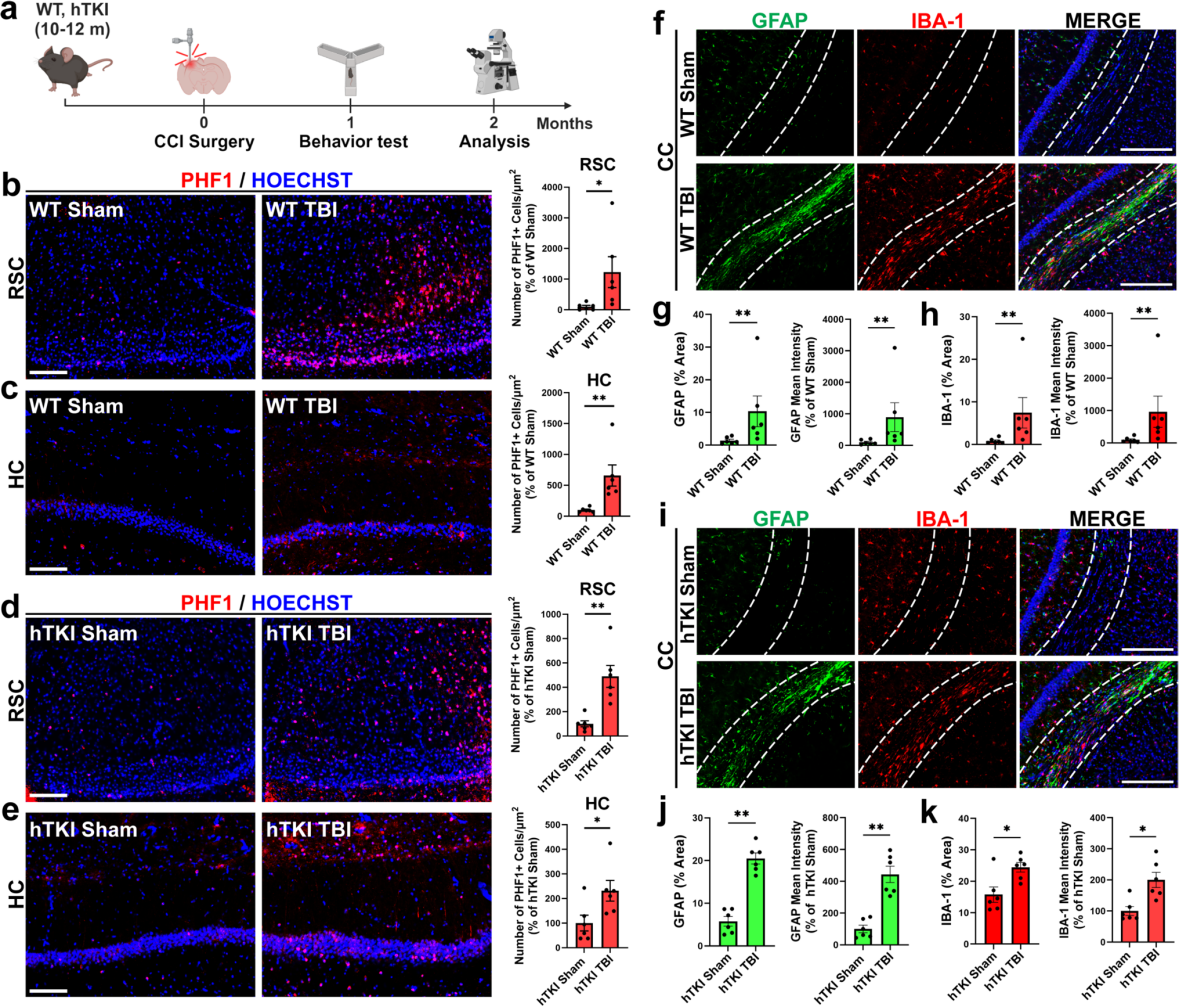
Single TBI increases tau hyperphosphorylation and gliosis in C57BL6/J (WT) and hTKI mice. **a** Experimental timeline of CCI TBI surgery, followed by behavioral testing and analysis (figure made with BioRender). **b**, **c** Left panel: Representative immunofluorescence (IF) images of PHF1 + (red) staining (**b**) in the retrosplenial cortex (RSC) and (**c**) the hippocampus (HC) region of WT Sham and WT TBI mice. Scale bar, 100 μm. Right Panel: The number of PHF1 + cells/µm^2^ was quantitated (**b**) in the RSC and (**c**) in the HC of WT Sham and WT TBI mice (*P < 0.05, **P < 0.01; Mann–Whitney test, *n* = 6 mice/group). **d**, **e** Left panel: Representative IF images of PHF1 + staining (**d**) in the RSC and (**e**) the HC region of hTKI Sham and hTKI TBI. Scale bar, 100 μm. Right Panel: The number of PHF1 + cells/µm^2^ was quantitated (**d**) in the RSC and (**e**) in the HC of hTKI Sham and hTKI TBI (*P < 0.05, **P < 0.01; Mann–Whitney test, *n* = 6 mice/group). **f** Representative IF images of GFAP (green) and IBA-1 (red) staining in the corpus callosum (CC) region of WT Sham and WT TBI mice. Scale bar, 100 μm. **g** (Left) Quantification of the % Area of GFAP and (Right) the mean intensity of GFAP in the CC region of WT Sham and WT TBI mice (**P < 0.01; Mann–Whitney test, *n* = 6 mice/group). **h** (Left) Quantification of the % Area of IBA-1 and (Right) the mean intensity of IBA-1 in the CC region of WT Sham and WT TBI mice (**P < 0.01; Mann–Whitney test, *n* = 6 mice/group). **i** Representative IF images of GFAP (green) and IBA-1 (red) in the CC region of hTKI Sham and hTKI TBI mice. Scale bar, 100 μm. **j** (Left) Quantification of the % Area of GFAP and (Right) the mean intensity of GFAP in the CC region of hTKI Sham and hTKI TBI mice (**P < 0.01; Mann–Whitney test, *n* = 6 mice/group). **k** (Left) Quantification of the % Area of IBA-1 and (Right) the mean intensity of IBA-1 in the CC region of hTKI Sham and hTKI TBI (*P < 0.05; Mann–Whitney test, *n = *6 mice/group). Nuclei for all images were counterstained by Hoechst 33342

Previous reports indicate that TBI increases tau pathology in several mouse models [23, 27, 44, 45, 66, 74, 91, 97, 107, 112]. Thus, we investigated the changes of ptau in WT (C57BL/6 J) and hTKI mice with a single TBI (induced cortical impact, CCI) compared to sham controls. We performed triple immunofluorescence (IF) staining with SATB2, OLIG2, and PHF1 (pSer396/pSer404) or CP13 (pSer202) and found that a single unilateral TBI increased ptau (PHF1-positive ( +) and CP13 +) in EX neurons (SATB2 +) and OLG (OLIG2 +) in the ipsilateral retrosplenial cortex (RSC) and hippocampus (HC) region of WT and hTKI mice compared with sham mice, respectively (Fig. 1b–e and Supplementary Fig. 2a–d). Notably, there was no co-localization between ptau and inhibitory (IN) neurons (GAD1 +), astrocytes (GFAP +), microglia/macrophages (IBA-1 +), or oligodendrocyte precursor cells (PDGFR-α+) (Supplementary Fig. 2f–i). Taken together, these results suggest that EX neurons and OLG are differentially vulnerable to ptau accumulation after TBI.

Aside from the accumulation of ptau in EX neurons and OLG, TBI has been shown to induce other pathologies, such as gliosis, synaptic dysfunction, and memory deficits [43, 59, 91, 104, 106, 118]. We sought to investigate changes of astro- and microgliosis in the RSC (cortical region proximal to the injury site), the corpus callosum (CC; white matter region proximal to injury site), and the lateral posterior nucleus of the thalamus (LP; a thalamic nucleus distal to the injury site) (Supplementary Fig. 3i) in WT and hTKI mice with TBI compared to sham controls. Using double IF staining with GFAP and IBA-1, we found that the immunoreactivity and the percentage of area occupied by GFAP and IBA-1 increased in the ipsilateral CC region of both mouse models with TBI compared to sham, indicating reactive gliosis in the white matter region proximal to the injury site (Fig. 1f–k). Furthermore, we found that the immunoreactivity and the percentage of area occupied by GFAP increased in the ipsilateral RSC region of WT and hTKI mice with TBI (Supplementary Fig. 3a, b, d, e). Microgliosis was also evident in the ipsilateral RSC region of WT and hTKI mice, as shown by the increase of IBA-1 immunoreactivity and percentage of area occupied by IBA-1 staining in WT mice with TBI (Supplementary Fig. 3a, c) while the surface area of IBA-1 + microglia was reduced in hTKI mice subjected to TBI (Supplementary Fig. 3d, f), compared to the sham controls. Additionally, we found that the immunoreactivity of GFAP and IBA-1 was increased in the ipsilateral LP of WT TBI and hTKI TBI mice compared to sham controls, indicating that deep brain structures distal from the site of injury are also affected by micro- and astrogliosis after TBI (Supplementary Fig. 3g, h). Overall, these results indicate that a single CCI TBI is sufficient to induce sustained glial activation in regions proximal and distal to the injury site **(**Fig. 1f–k and Supplementary Fig. 3a–h).

**Fig. 2:**
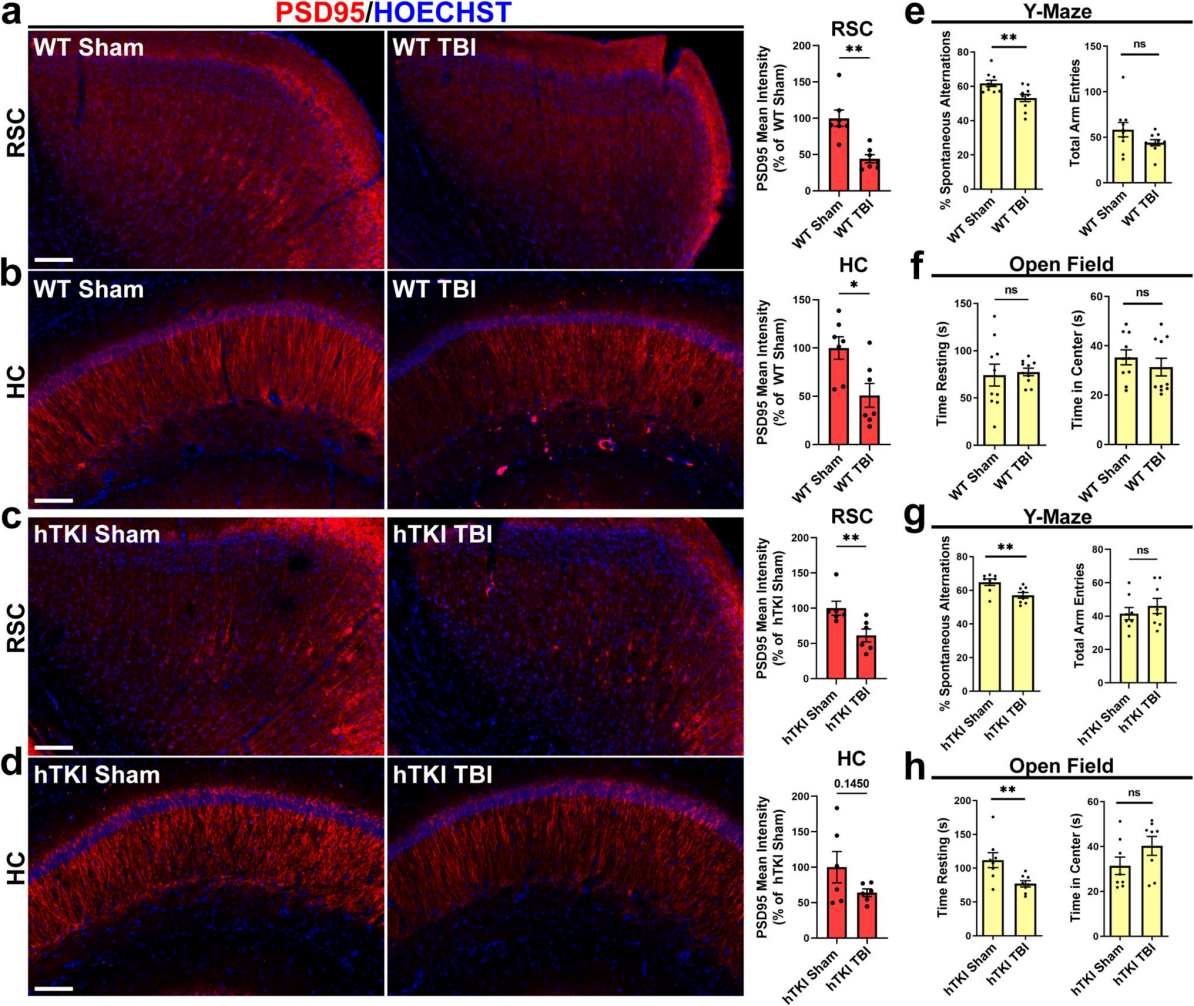
TBI reduces postsynaptic density protein 95 (PSD95) and induces memory deficits in WT and hTKI mice. **a**, **b** Left Panel: Representative IF images of PSD95 (red) immunoreactivity in (**a**) the RSC and (**b**) the HC of WT Sham and WT TBI mice. Scale bar, 100 μm. Right Panel: Quantification of the mean intensity of PSD95 in (**a**) the RSC and (**b**) the HC of WT Sham and WT TBI mice (*P < 0.05 and **P < 0.01; Mann–Whitney test, *n* = 7 mice/group). **c**, **d** Left Panel: Representative IF images of PSD95 (red) immunoreactivity in (**c**) the RSC and (**d**) the HC of hTKI Sham and hTKI TBI mice. Scale bar, 100 μm. Right Panel: Quantification of the mean intensity of PSD95 in (**c**) the RSC and (**d**) in the HC of hTKI Sham and hTKI TBI mice (**c** **P < 0.01 and **d** *P* = 0.1450; Mann–Whitney test, *n* = 6 mice/group). Nuclei for all images were counterstained by Hoechst 33342. **e**, **g** Left Panel: The % of spontaneous alternations (actual alternations/possible alternations (total arm entries—2) × 100) in the Y-maze was used to assess spatial working memory performance 1 month following TBI in (**e**) WT Sham vs WT TBI mice and (**g**) hTKI Sham vs hTKI TBI mice (**P < 0.01; Mann–Whitney test, *n* = 8–10 mice/group). Right Panel: The number of total arm entries was used to confirm a similar amount of movement in the Y-maze in (**e**) WT Sham vs WT TBI mice and (**g**) hTKI Sham vs hTKI TBI mice (Mann–Whitney test, *n* = 8–10 mice/group). **f**, **h** Left Panel: The total time spent resting was measured to evaluate the exploratory and locomotor activity in (**f**) WT Sham vs WT TBI mice and (**h**) hTKI Sham vs hTKI TBI mice (**P < 0.01; Mann–Whitney test, *n* = 8–10 mice/group). Right Panel: The total time spent in the center was used as a measure of the exploratory and locomotor activity in (**f**) WT Sham vs WT TBI mice and (**h**) hTKI Sham vs hTKI TBI mice (Mann-Whiney test, *n* = 8–10 mice/group). *ns* non-significant

Previous reports indicate that both tau pathology and reactive gliosis contribute to synaptic dysfunction and memory deficits [23, 29, 40, 51, 57, 91, 101, 104]. We sought to investigate synaptic integrity after TBI compared to sham controls. To assess synaptic function after TBI, we used IF staining with a specific antibody against postsynaptic density protein 95 (PSD95, marker of post-synaptic density) or synaptosome associated protein 25 (SNAP-25, marker of pre-synaptic function). Notably, we found that TBI induced post-synaptic dysfunction in the ipsilateral RSC and hippocampus regions in both WT and hTKI mouse models as shown by decreased PSD95 immunoreactivity (Fig. 2a–d), but TBI did not alter pre-synaptic function as shown by similar SNAP-25 immunoreactivity (Supplementary Fig. 4a–d), compared to sham mice, respectively. Given that synaptic dysfunction contributes to cognitive deficits [29, 36, 51, 108], we further analyzed cognitive function and the exploratory and locomotor activity after TBI using Y-maze and the open-field test, respectively. In agreement with the observed post-synaptic dysfunction, WT and hTKI mice exhibited worsened performance on the Y-maze following TBI as shown by a decrease in the number of spontaneous alternations in both TBI groups, indicating worsened spatial working memory (Fig. 2e, g). Additionally, hTKI TBI mice showed altered performance on the open-field test, spending less time resting than sham controls (Fig. 2h). Taken together, these results indicate that the CCI mouse model of TBI results in ptau accumulation, gliosis, and memory deficits in WT and hTKI mice compared to sham controls, indicating that this model is sufficient to investigate the molecular mechanisms that underlie the observed pathological changes.

### Tau hyperphosphorylation and gliosis in human brains

**Fig. 3:**
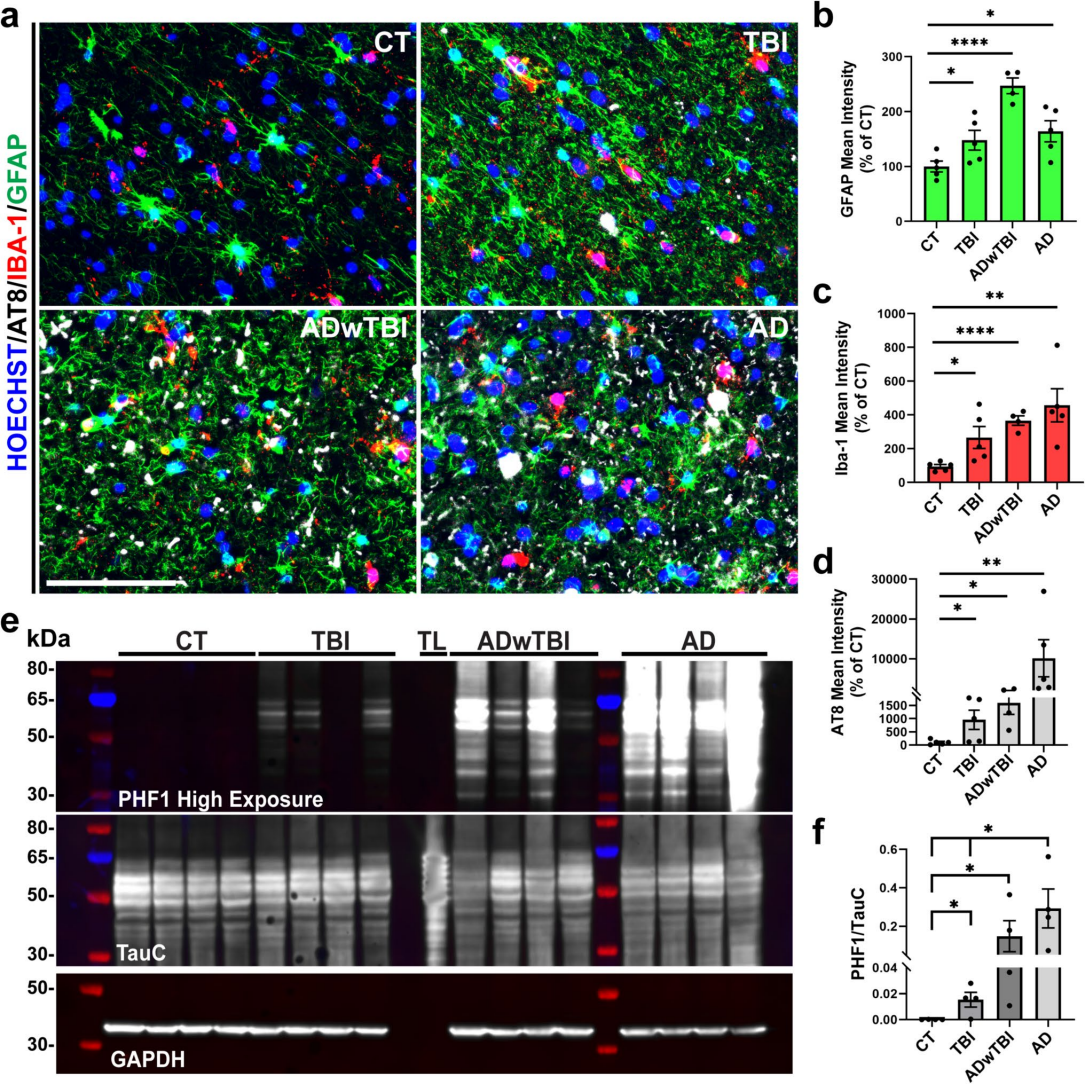
TBI and AD increase tau hyperphosphorylation and gliosis in the inferior parietal lobe (IPL) of human brains. **a** Representative IF images of GFAP (green), IBA-1 (red), and AT8 (white) immunoreactivity in the IPL of post-mortem human brain tissue (CT, TBI, AD with TBI history (ADwTBI), and AD without TBI history (AD)). Scale bar, 100 μm. **b**, **c** Quantification of the mean intensity of (**b**) GFAP and (**c**) IBA-1 in the IPL (*P < 0.05, **P < 0.01, ****P < 0.0001 vs CT; unpaired *t*-test, *n* = 4–5 cases/group). **d** Quantification of the mean intensity of AT8 in the IPL (*P < 0.05, **P < 0.01 vs CT; unpaired *t*-test, *n* = 4–5 cases/group). Nuclei for all images were counterstained by Hoechst 33342. **e** The total protein lysates from the IPL of CT, TBI, ADwTBI, and AD cases were subjected to Western blot (WB) assay with specific antibodies against mouse anti-PHF1 (top), rabbit anti-TauC (middle), and rabbit anti-GAPDH (bottom, loading control). TL: recombinant tau ladder with 6 isoforms of tau protein. The isoforms are in order: 2N4R, 2N3R, 1N4R, 1N3R, 0N4R, 0N3R. **f** Quantification of the ratio of PHF1/TauC (*P < 0.05 TBI vs CT, ADwTBI vs CT, AD vs CT, and AD vs TBI; Mann–Whitney test, *n* = 4 cases/group). Full-length western blots can be visualized in Supplementary Fig. 10

To validate the pathological profiles of TBI identified in animal models, we sought to explore the pathological profiles of post-mortem human brain tissue from control (CT), TBI, ADwTBI (AD with a history of TBI), and AD (AD without a history of TBI) using IF and western blot (WB) in the inferior parietal lobe (IPL). Previous reports suggest that the parietal lobe is frequently injured in human TBI cases and continues to atrophy for one-year post-injury [11, 64, 79]. In accordance with the TBI mouse models, post-mortem tissue from TBI cases revealed higher ptau at several phospho-epitopes (PHF1 (pSer396/pSer404) and AT8 (pSer202/pThr205)) compared to controls **(**Fig. 3a, d–f and Supplementary Fig. 5h), while ADwTBI exhibited even higher tau pathology (Fig. 3e, f). To better understand the type of pathology in the human brain samples, we characterized the PHF1 + cells and number of PHF1 + neuritic plaques across the different disease conditions. Notably, the cases with a history of TBI showed a higher number of PHF1 + cells and neuritic plaques compared to the control cases, while the presence of these two types of pathology was even higher in ADwTBI and AD cases (Supplementary Fig. 5g–j).

Furthermore, using IF staining, we examined reactive gliosis in all disease conditions, revealing an interesting phenotype. TBI, ADwTBI, and AD cases all show substantial astrogliosis (increased GFAP immunoreactivity) and microgliosis (increased IBA-1 immunoreactivity) compared with controls (Fig. 3a–c). Despite a higher ptau load in AD, the amount of gliosis is similar in TBI, indicating that TBI may significantly increase neuroinflammation apart from tau pathology. Thus, as many studies indicate [93], the role of neuroinflammation after TBI is a pertinent aspect of the pathological profile. Although globally cases of TBI experience robust gliosis compared to controls, we were interested to know whether gliosis changed more dramatically in regions burdened with tau pathology. We found that the number of IBA1 + microglia and GFAP + astrocytes increased as the percentage area occupied by PHF1 + tau pathology increased across all disease conditions, suggesting that microglia and astrocytes are recruited to regions with tau pathology (Supplementary Fig. 5a–f). Overall, these data indicate that the pathological profiles of post-mortem human brain tissue in all disease conditions are similar to those observed in both WT and hTKI mice subjected to CCI TBI. Patients with a history of TBI and AD show higher tau pathology and gliosis compared to controls, providing motivation for determining the mechanism underlying these pathological changes although the selection of human TBI cases has a potential bias due to the limited number of TBI cases available in the Banner Sun Health Research Institute Brain and Body Donation Program.

### BAG3 decreases in neurons and OLG accumulated with ptau, while it increases in astrocytes in both wild-type and hTKI mice after TBI as well as in the human brain

**Fig. 4:**
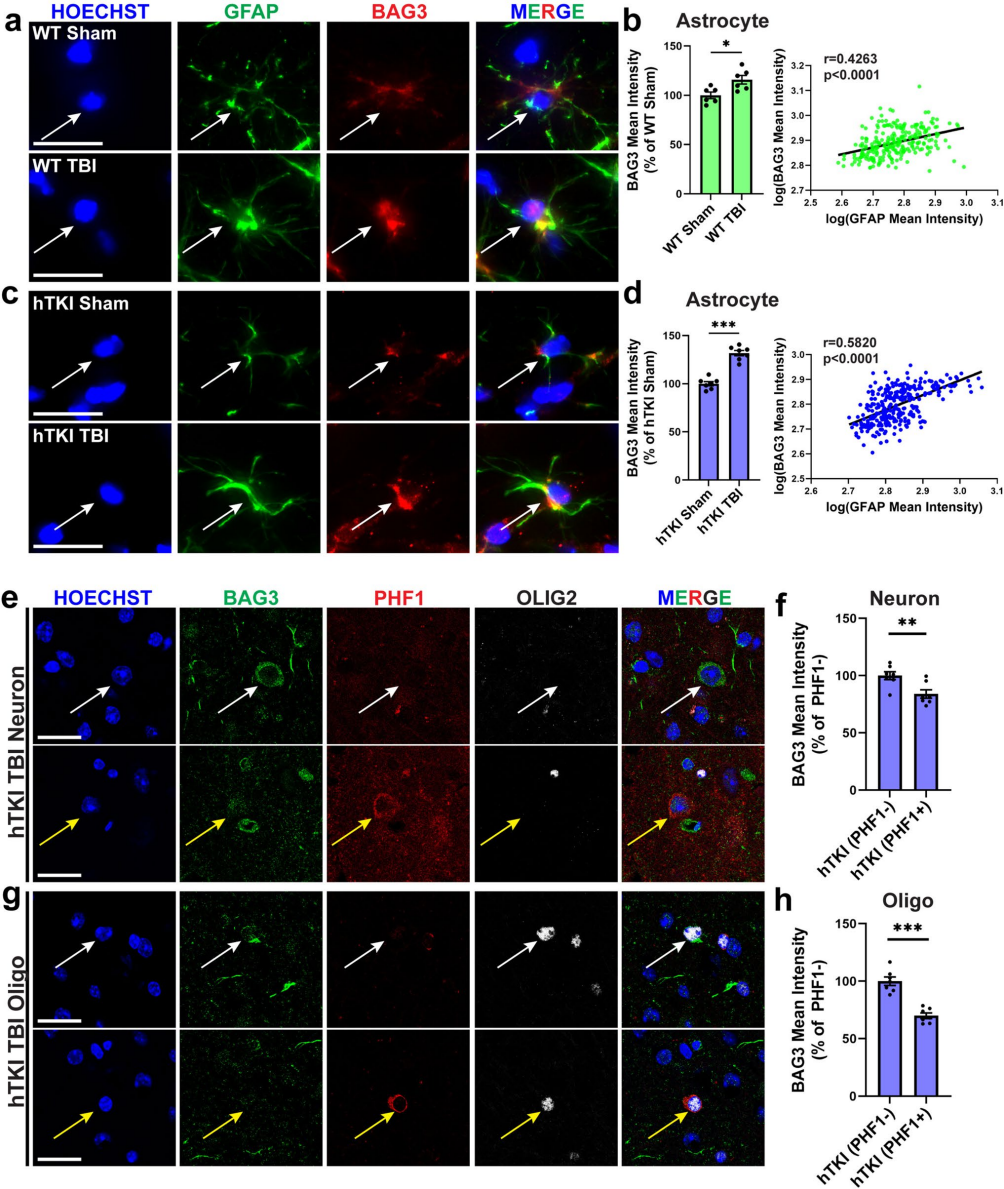
TBI increases BAG3 immunoreactivity in astrocytes in WT and hTKI mice while BAG3 immunoreactivity decreases in neurons and OLG with ptau accumulation. **a**, **c** Representative epifluorescence IF images of GFAP (green) and BAG3 (red) immunoreactivity in the CA1 region of (**a**) WT Sham and WT TBI, (**c**) hTKI Sham and hTKI TBI mice. Scale bar, 20 μm. **b**, **d** Left Panel: Quantification of the mean intensity of BAG3 in GFAP + astrocytes in (**b**) WT Sham vs WT TBI mice and (**d**) hTKI Sham vs hTKI TBI mice (*P < 0.05, ***P < 0.001; Mann–Whitney test, *n* = 6–7 mice/group, average of 20–23 cells/mouse). Right Panel: Correlation of log_10_(GFAP Mean Intensity) and log_10_(BAG3 Mean Intensity) in (**b**) WT Sham and WT TBI mice and in (**d**) hTKI Sham and hTKI TBI mice (*P* < 0.0001; Spearman Correlation: **b** *r* = 0.4263, *n* = 244, **d** *r* = 0.5820, *n* = 291). **e**, **g** Representative confocal IF images of BAG3 (green), PHF1 (red), and OLIG2 (white) immunoreactivity in the cortex of hTKI TBI mice. Yellow arrows indicate (**e**) PHF1 + neurons (PHF1 + /OLIG2-) and (**g**) PHF1 + oligodendrocytes (PHF1 + /OLIG2 +). White arrows indicate (**e**) PHF1- neurons (PHF1-/OLIG2-) and (**g**) PHF1- oligodendrocytes (PHF1-/OLIG2 +). Scale bar, 20 μm. **f, h** Quantification of the mean intensity of BAG3 in (**f**) PHF1 + vs PHF1- neurons of hTKI TBI mice and in (**h**) PHF1 + and PHF1- oligodendrocytes in hTKI TBI mice (**P < 0.01, ***P < 0.001; Mann–Whitney test, *n* = 7 mice/group, average of 10 cells/mouse). Nuclei for all images were counterstained by Hoechst 33342

**Fig. 5:**
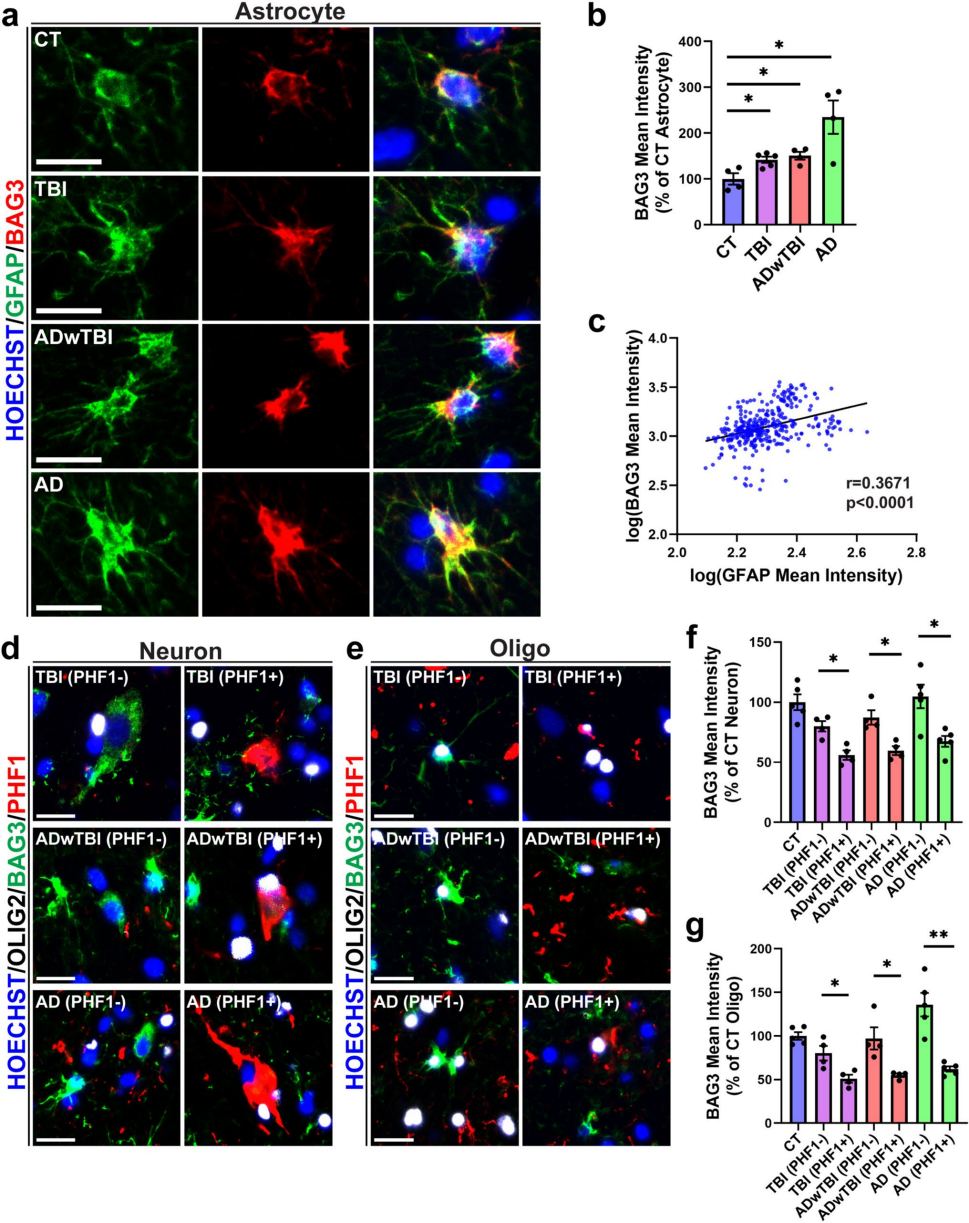
TBI and AD increases BAG3 immunoreactivity in astrocytes while BAG3 immunoreactivity decreases in neurons and OLG with ptau accumulation in the IPL of human postmortem brains. **a** Representative IF images of GFAP (green) and BAG3 (red) immunoreactivity in the IPL of CT, TBI, ADwTBI, and AD. Scale bar, 20 μm. **b** Quantification of the mean intensity of BAG3 in GFAP + cells (*P < 0.05 vs CT; Mann–Whitney test, n = 4–5 cases/group, average of 20–21 cells/case). **c** Correlation of log_10_(GFAP Mean Intensity) and log_10_(BAG3 Mean Intensity) with combined human cases (*P* < 0.0001; Spearman Correlation: *r* = 0.3671, *n* = 342). **d**, **e** Representative IF images of BAG3 (green), PHF1 (red), and OLIG2 (white) immunoreactivity in the IPL in (**d**) neurons and (**e**) OLG across the human conditions. Scale bar, 20 μm. **f**, **g** Quantification of the mean intensity of (**f**) PHF1 + neurons and (**g**) PHF1 + OLG in TBI, ADwTBI, and AD compared to (**f**) PHF1- neurons and (**g**) PHF1- OLG in the respective disease (*P < 0.05, **P < 0.01; Mann–Whitney test, *n* = 4–5 cases/group, average of 7–10 cells/case). Nuclei for all images were counterstained by Hoechst 33342

Previously, we reported that BAG3 is reduced in human EX neurons vulnerable to tau accumulation and increased in astrocytes resistant to tau accumulation [22]. Furthermore, previous studies implicate BAG3 in pathological tau clearance in neurons [42, 49, 52–54], and others have also shown that astrocytic upregulation of BAG3 can help facilitate the clearance of tau and alpha-synuclein aggregates [90]. Despite the thorough investigation between tau and BAG3, little is known about the cell type-specific alterations of BAG3 after TBI and its relationship with tau pathology. Using IF staining with specific antibodies against BAG3, GFAP, Olig2, and PHF1, we found that a single unilateral TBI enables changes in the protein level of BAG3 in neurons, OLG, and astrocytes in both WT and hTKI mice (Fig. 4 and Supplementary Fig. 6). The protein level of BAG3 was significantly increased in GFAP + astrocytes with TBI compared to sham controls (Fig. 4a–d). Thus, astrocytes may upregulate BAG3 as a compensatory mechanism, to protect themselves from tau accumulation (Supplementary Fig. 2g) or to facilitate the clearance of tau aggregates. Along with the findings from mouse models of TBI, the levels of astrocytic BAG3 also increase in human cases with TBI, ADwTBI, and AD (Fig. 5a, b). Notably, we observed a positive correlation between the reactive astrocyte marker GFAP and BAG3 in the mouse models of TBI (Fig. 4b, d) and in post-mortem brain tissue (Fig. 5c). Thus, astrocytic BAG3 may be considered as a promising marker of disease-associated astrocytes across a variety of diseases [90]**.**

We further investigated how BAG3 changed in PHF1 + neurons and PHF1 + OLG compared to their PHF1-negative (-) counterparts in mouse models of TBI and post-mortem brain tissue. We found a consistent trend, showing an inverse relationship between BAG3 and PHF1 levels at single cell levels, i.e., cells with accumulation of ptau have significantly reduced BAG3 compared to cells without ptau accumulation (Fig. 4e–h, Supplementary Fig. 6, Fig. 5d–g, and Supplementary Fig. 7) although global BAG3 increased in animal models and human post-mortem brain tissues with TBI and AD (Supplementary Fig. 8). Thus, accumulation of ptau may be related to deficient levels of BAG3 and its pre-established role in the ALP and clearance of protein aggregates [42, 49, 52–54]. To our knowledge, this is the first time BAG3 deficiency may be a contributing factor to ptau accumulation in oligodendrocytes after TBI or in AD. Taken together, these results suggest that a single TBI can alter the cell-type-specific expression of BAG3 in neurons, OLG, and astrocytes in mouse models of TBI and post-mortem human tissue, making cells more or less vulnerable to ptau accumulation after TBI.

### Overexpression of neuronal BAG3 reduces tau hyperphosphorylation, synaptic dysfunction, and cognitive deficits in hTKI mice induced by TBI

**Fig. 6:**
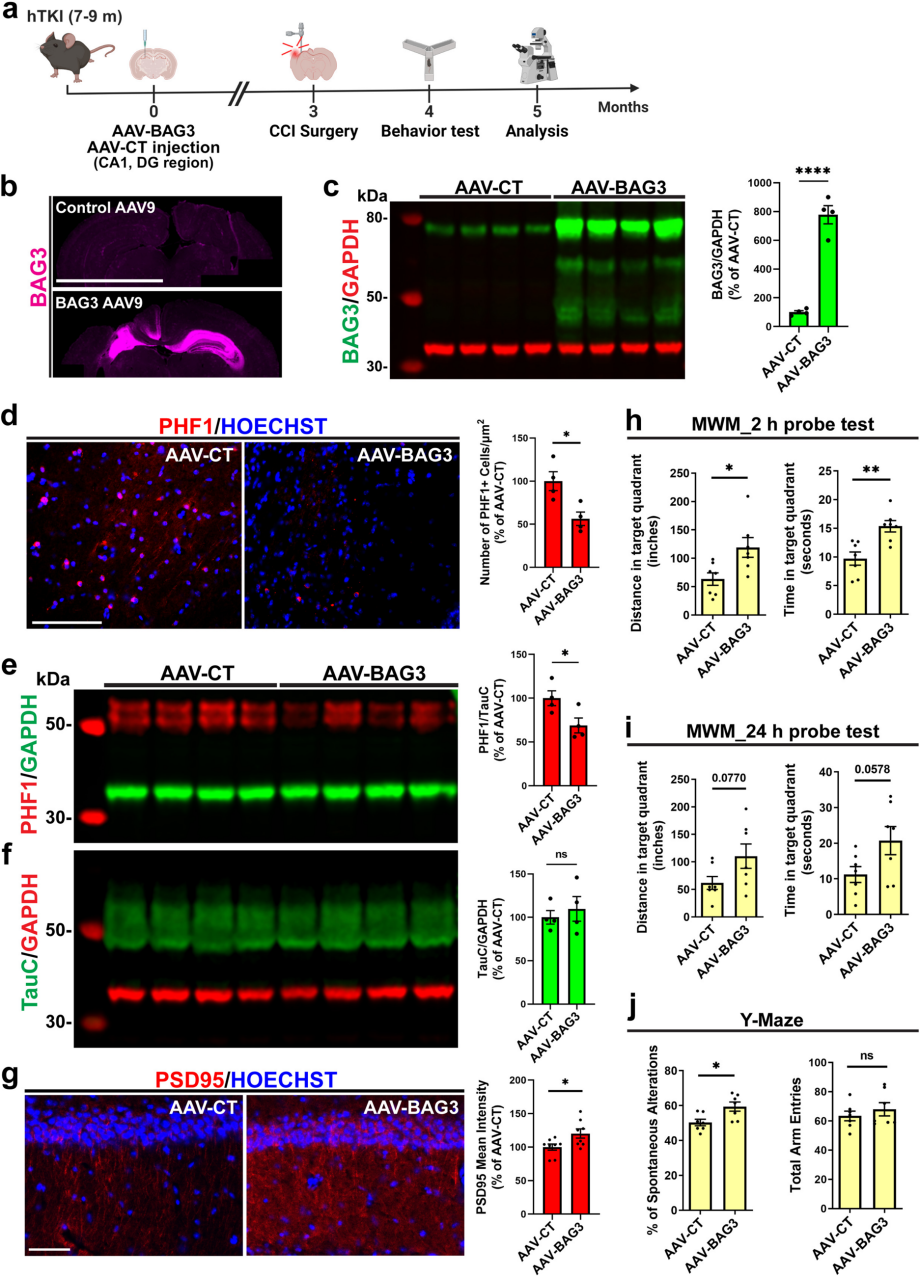
Overexpression of neuronal BAG3 reduces tau hyperphosphorylation, synaptic dysfunction, and cognitive deficits in hTKI mice induced by TBI. **a** Experimental timeline of AAV9-BAG3 or AAV9-GFP (CT) injection followed by CCI TBI (figure made with BioRender). **b** Representative IF images of BAG3 overexpression (Magenta) after AAV9-BAG3 injection compared to AAV9-GFP control injection in hTKI mice. Scale bar, 4 mm. **c** Left Panel: hTKI mouse lysate from the hippocampus and cortex regions were collected and subjected to WB assay with a rabbit anti-BAG3 and mouse anti-GAPDH specific antibody. Right Panel: Quantification from WB assay to confirm BAG3 overexpression in AAV-BAG3-injected mice compared to AAV-CT (****P < 0.0001; unpaired *t*-test, *n* = 4 mice/group). **d** Left Panel: Representative IF staining of PHF1 (red) from the intra-CA2–CA3 region of HC. Scale bar, 100 μm. Right Panel: Quantification of the number of PHF1 + cells/µm^2^ in the CA2–CA3 region of HC (*P < 0.05; unpaired *t*-test, *n* = 4 mice/group). **e**, **f** Left Panel: Mouse lysate from the hippocampus and cortex regions were collected and subjected to WB assay with (**e**) mouse anti-PHF1 and rabbit anti-GAPDH specific antibodies or (**f**) rabbit anti-TauC and mouse anti-GAPDH specific antibodies. Right Panel: Quantification from WB assay to determine the ratio of (**e**) PHF1/TauC or (**f**) TauC/GAPDH in AAV-CT vs AAV-BAG3-injected mice (*P < 0.05; unpaired *t*-test, *n* = 4 mice/group). **g** Left Panel: Representative IF images of PSD95 (red) in the HC of AAV-CT and AAV-BAG3 mice. Scale bar, 50 μm. Right Panel: Quantification of PSD95 mean intensity from the HC of AAV-CT vs AAV-BAG3 mice (*P < 0.05; Mann–Whitney test, *n* = 4 mice/group, 2 image/mouse). **h**, **i** Morris Water Maze behavioral test to assess the (**h**) short-term (2 h) and (**i**) long-term (24 h) spatial learning and memory in AAV-CT and AAV-BAG3-injected mice subjected to TBI. (Left) Measurement of the distance and (Right) time the mice remained in the target quadrant (*P* = 0.0578, *P* = 0.0770, *P < 0.05, and **P < 0.01; unpaired *t*-test, *n* = 7 mice/group). **j** Y-maze behavioral test to evaluate spatial working memory performance in AAV-BAG3 compared to AAV-CT injected mice after TBI. (Left) The percentage of spontaneous alternations was calculated by (actual alternations/possible alternations (total arm entries – 2) × 100)). (Right) The total number of arm entries was used to confirm similar levels of movement between groups in the Y-Maze (*P < 0.05; unpaired *t*-test, *n* = 7 mice/group). Nuclei for IF images were counterstained by Hoechst 33342. ns; non-significant. Full-length western blots can be visualized in Supplementary Fig. 10

The deficient level of BAG3 in neurons and OLG with ptau accumulation encouraged us to overexpress BAG3 in hippocampal neurons prior to TBI, to determine if increasing BAG3 level in neurons is sufficient to attenuate pathology induced by TBI in hTKI mice. To overexpress BAG3 in hippocampal neurons, we used stereotaxic unilateral microinjection of AAV9-hSYN1-eGFP-2A-hBAG3-WPRE (AAV-BAG3) or control AAV9-hSYN1-eGFP-2A-WPRE (AAV-CT) into the hippocampal CA1 and dentate gyrus (DG) region of 7–9-month-old hTKI mice. These mice were subjected to TBI surgery 3 months after the virus injection, and behavior tests and following experiments were performed 1-month post-injury (Fig. 6a). We confirmed the BAG3 overexpression in the hippocampus region of hTKI mice by BAG3 antibody using IF staining and WB assay (Fig. 6b, c). Notably, we found that overexpression of neuronal BAG3 can reduce the number of cells with ptau accumulation in the CA2–CA3 region of the hippocampus compared to AAV-CT (Fig. 6d and Supplementary Fig. 2e). Additionally, global levels of ptau were reduced in BAG3 overexpressed mice compared to AAV-CT, shown by significantly reduced ratios of PHF1/TauC (total tau) via WB assay (Fig. 6e, f), indicating that neuronal BAG3 overexpression can significantly reduce the global ptau burden.

We also compared the presence of gliosis in AAV-BAG3 mice to AAV-CT mice using IF staining. Surprisingly, we found no significant differences in the amount of gliosis in the ipsilateral CC or the hippocampus region of BAG3-injected mice compared to control mice (Supplementary Fig. 9a, b). These results suggest that neuronal BAG3 cannot prevent gliosis that is significantly impacted after TBI although it can prevent ptau accumulation. These results also indicate that TBI-induced gliosis does not necessarily coincide with the severity of ptau accumulation, and TBI may induce gliosis in parallel to ptau accumulation. Similar to our observations of no further increase of gliosis in human cases with AD compared to human TBI-only cases, mouse models of TBI experience ramped activation of astrocytes and microglia, which cannot be rescued by overexpression of BAG3 in neurons specifically, even though ptau accumulation is reduced.

Furthermore, we wanted to investigate if overexpression of BAG3 in neurons can prevent synaptic dysfunction and memory deficits induced by TBI. We assessed the immunoreactivity of PSD95, SNAP-25, and memory in AAV-BAG3 and AAV-CT mice. Mice injected with AAV-BAG3 experienced increased hippocampal PSD95 (Fig. 6g), while SNAP-25 remained unchanged (Supplementary Fig. 9c). Mice with AAV-BAG3 had improved performance in short-term and long-term memory on the Morris water maze cognitive test, showing improvement on recalling the goal quadrant (Fig. 6h, i), and in the Y-maze, showing an increased percentage of spontaneous alternations (Fig. 6j), compared to the AAV-CT mice. Our results suggest that overexpression of neuronal BAG3 is a promising strategy to reduce the burden of ptau and attenuate memory deficits after TBI; however, overexpression of BAG3 in the neurons alone is not sufficient to reduce the gliosis observed following TBI.

### Autophagy dynamics are regulated by the level of BAG3 in vitro

**Fig. 7:**
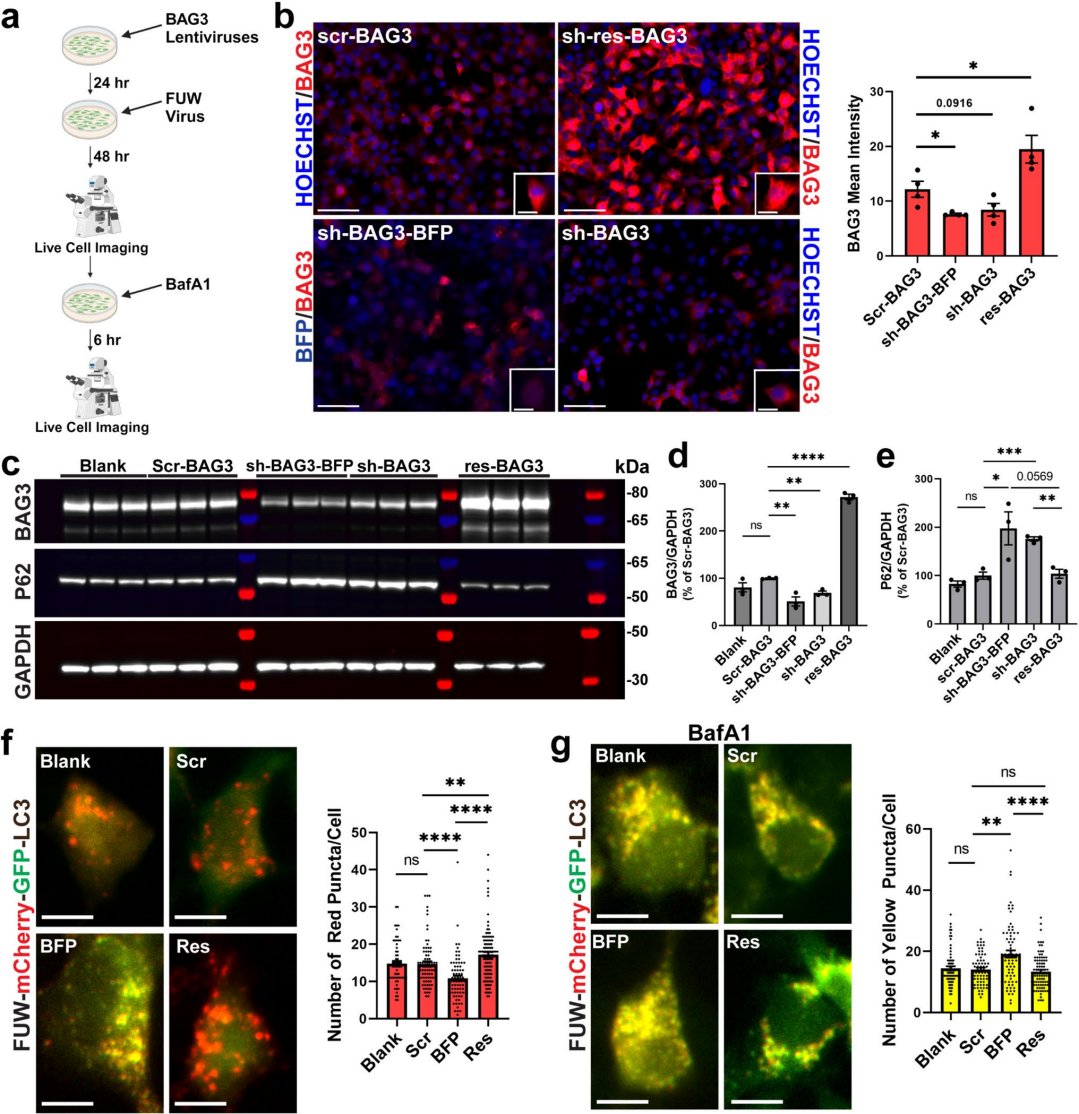
Overexpression of BAG3 promotes autophagy flux, while knockdown of BAG3 inhibits it. **a** The experimental timeline of FUW mCherry-GFP-LC3 virus treatment in BAG3 modulated HEK293 cells. **b** Left Panel: Representative IF images of HEK293 cells after 48 h of treatment with various BAG3 lentiviruses (scr-BAG3, sh-res-BAG3, sh-BAG3-BFP, and sh-BAG3) using a rabbit anti-BAG3 specific antibody. Scale bar, 50 μm (low-magnification images), 10 μm (inset images). Nuclei for IF images were counterstained by Hoechst 33342. Right Panel: Quantification of BAG3 mean intensity after lentivirus treatment (*P* = 0.0916, *P < 0.05 vs scr-BAG3; unpaired *t*-test, *n* = 4 replicates/group). **c** Protein lysate was extracted from HEK293 cells and subjected to WB assay with (top) rabbit anti-BAG3, (middle) mouse anti-p62, and (bottom) rabbit anti-GAPDH. **d** Quantification of the ratio of BAG3/GAPDH to confirm knock-down or overexpression of BAG3 after lentivirus treatment (**P < 0.01, ****P < 0.0001 vs scr-BAG3; unpaired *t*-test, *n* = 3 replicates/group). **e** Quantification of the ratio of p62/GAPDH to assess autophagic flux after lentivirus treatment (*P < 0.05, ***P < 0.001 vs scr-BAG3; *P* = 0.0569 sh-BAG3-BFP vs res-BAG3; **P < 0.01 sh-BAG3 vs res-BAG3; unpaired *t*-test, *n* = 3 replicates/group). **f** Left Panel: Representative images of live HEK293 cells after treatment with BAG3 and FUW lentiviruses. Yellow puncta (green and red double positive) indicate phagosomes/autophagosomes, and single red puncta indicates autolysosome formation after the GFP signal is quenched by the acidic lysosome. Scale bars, 10 μm. Right Panel: Quantification of the number of single red puncta per cell after modulating BAG3 (****P < 0.0001 Scr vs BFP; **P < 0.01 Res vs Scr; ****P < 0.0001 BFP vs Res; Mann–Whitney test, *n* = 3 replicates/group, 20–46 cells/replicate). **g** Left Panel: Representative images of live HEK293 cells after treatment with BafA1, an autophagy flux inhibitor. Yellow puncta indicate phagosome/autophagosomes. Scale bars, 10 μm. Right Panel: Quantification of the number of yellow (green and red double positive) puncta per cell after modulating BAG3 and treating with BafA1 (**P < 0.01 Scr vs BFP; ****P < 0.0001 BFP vs Res; Mann–Whitney test, *n* = 3 replicates/group, 18–43 cells/replicate). *ns* non-significant. Full-length western blots can be visualized in Supplementary Fig. 10

Building upon prior research demonstrating BAG3’s role in facilitating macroautophagy and potentially reducing the accumulation of protein aggregates [12, 42, 49, 52–54, 90], coupled with our findings regarding the relationship between tau and BAG3, we were encouraged to question how modulation of BAG3 level impacted the autophagic flux in vitro using HEK293 cells. Using different lentiviral vectors [22, 49], HEK293 cells were transduced with scramble BAG3 control, BAG3 knock-down (sh-BAG3 and sh-BAG3-BFP), or BAG3 overexpression (shRNA-resistant BAG3, sh-res-BAG3) lentiviruses to modulate the level of BAG3 (Fig. 7a). After 48 h, the expressions of BAG3 after different lentivirus treatments were validated by IF staining and WB assay with an antibody against BAG3 (Fig. 7b–d). After validation of our constructs, we investigated the role of BAG3 on the autophagic flux by two methods: (1) analysis by WB assay using p62, a known substrate of autophagy degradation and a marker for autophagic flux [82]; and (2) using LC3 reporter virus, FUW mCherry-GFP-LC3, to visualize free autophagosome (GFP and mCherry fluorescence), autolysosome (mCherry only, due to acid sensitivity of GFP), and fusion of the autophagosome with lysosome. We found that knock-down of BAG3 (sh-BAG3-BFP, sh-BAG3) resulted in the accumulation of p62 (Fig. 7c, e) using the first method, suggesting that reduction in the level of BAG3 causes autophagic flux dysfunction. Also, overexpression of BAG3 (sh-res-BAG3) reduced p62 accumulation compared to the knock-down of BAG3 (Fig. 7c, e).

To further measure the effect of BAG3 on autophagy dynamics in living cells, HEK293 cells were transiently transduced with either BAG3 knock-down or overexpression lentiviruses, followed by the treatment of the FUW virus (Fig. 7a). Notably, BAG3 knock-down (sh-BAG3-BFP) significantly reduced the number of single red LC3 puncta, indicating impairment of the autophagic flux. Additionally, overexpression of BAG3 (sh-res-BAG3) increased the number of single red LC3 puncta compared to knock-down of BAG3 or scramble controls (scr-BAG3), suggesting that BAG3 overexpression can increase the efficiency of the autophagic flux (Fig. 7f). Subsequently, we conducted a similar experiment, with the administration of Bafilomycin-A1 (BafA1), a known inhibitor of autophagic flux by disruption of lysosomal acidification and autophagosome-lysosome fusion [63]. Under BafA1 conditions, modulation of BAG3 level still impacted the autophagic flux, indicating that BAG3 influences the ALP at several time points. Reduction of the BAG3 level (sh-BAG3-BFP) was sufficient to increase the number of yellow (green and red double positive) LC3 puncta, suggesting a further block of the ALP in addition to BafA1. Furthermore, overexpression of BAG3 (sh-res-BAG3) reduced the levels of yellow puncta to a similar level as the scramble BAG3 virus (scr-BAG3) (Fig. 7g). The results from FUW assay also suggest that increased p62 (Fig. 7c, e) is probably due to decreased p62 degradation via the block of autophagy flux by BAG3 knock-down, but not the upregulation of p62 transcription. Taken together, our results indicate that BAG3 is a potential modulator of the ALP, and therapeutic interventions targeting BAG3 may be sufficient to rescue dysfunction of the ALP seen in several neurodegenerative diseases with proteinaceous inclusions [10, 67].

### Autophagy-lysosome pathway (ALP) is inhibited in both wild-type and hTKI mice after TBI as well as human brains with TBI and/or AD history, while overexpression of BAG3 in neurons prevents it in hTKI mice

**Fig. 8:**
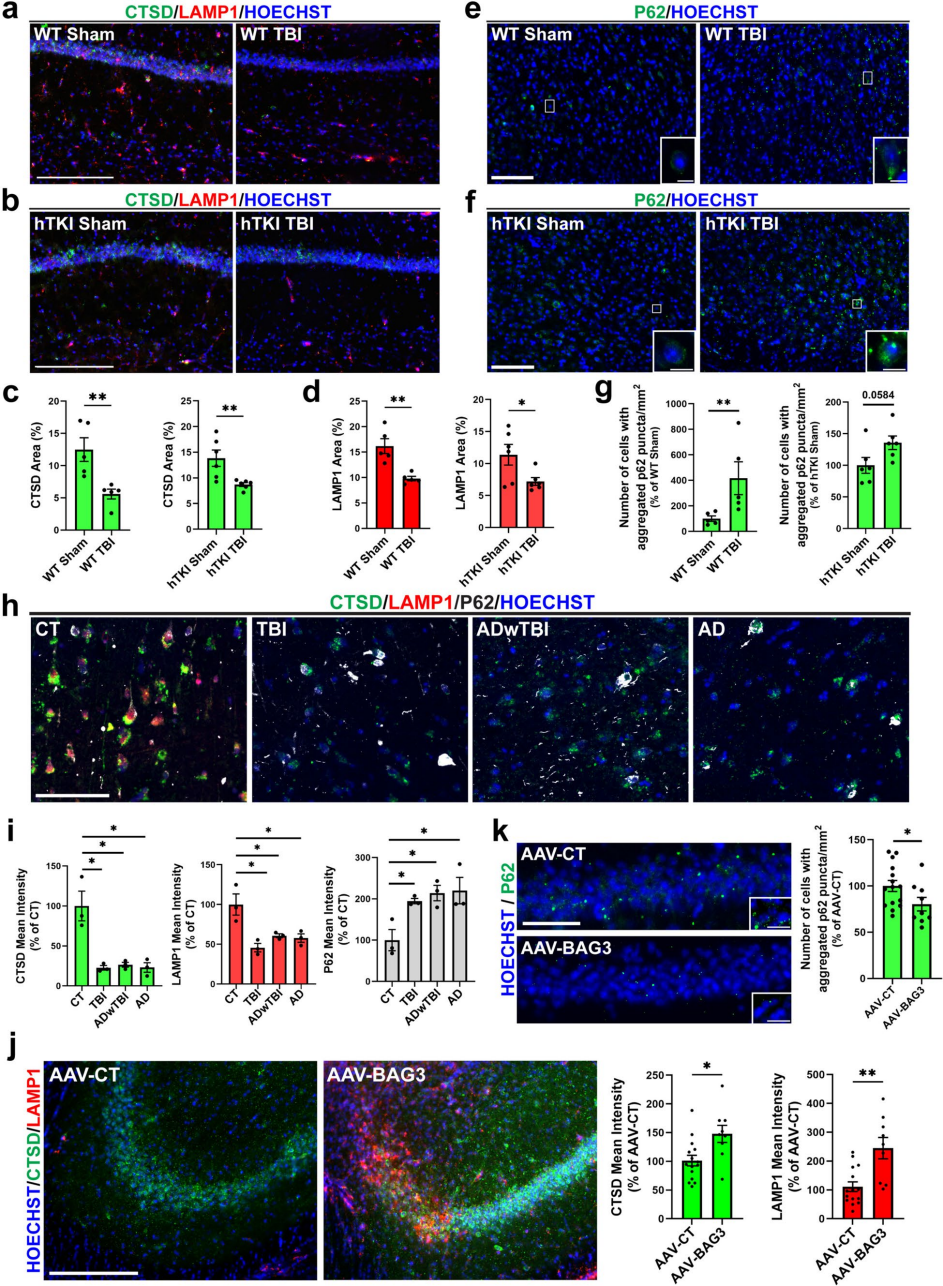
TBI reduces ALP-associated genes (CTSD and LAMP1) and increases p62 puncta formation in mouse models, consistent with human TBI and AD cases. Overexpression of BAG3 in neurons in hTKI mice prevents these changes. **a**, **b** Representative images of IF staining of CTSD (green) and LAMP1 (red) in the CA1 region of the hippocampus in (**a**) WT Sham and WT TBI, (**b**) hTKI Sham and hTKI TBI mice. Scale bar, 200 μm. **c** Quantification of the % Area of CTSD in (Left) WT Sham vs WT TBI mice and (Right) hTKI Sham vs hTKI TBI mice in the CA1 region of the hippocampus (**P < 0.01; unpaired *t*-test, *n* = 5–6 mice/group). **d** Quantification of the % Area of LAMP1 in (Left) WT Sham vs WT TBI mice and (Right) hTKI Sham vs hTKI TBI mice in the CA1 region of the hippocampus (*P < 0.05, **P < 0.01; unpaired *t*-test, *n* = 5–6 mice/group). **e**, **f** Representative images of IF staining of p62 (green) in the RSC of **(e)** WT Sham and WT TBI, (**f**) hTKI Sham and hTKI TBI mice. Scale bar, 100 μm (low-magnification images), 10 μm (inset images). **g** Quantification of the number of cells with aggregated p62 puncta/mm^2^ in (Left) WT Sham vs WT TBI mice and (Right) hTKI Sham vs hTKI TBI mice ((Left) **P < 0.01; Mann–Whitney test, *n* = 5 mice/group, (Right) *P* = 0.0584; unpaired *t*-test, n = 6 mice/group). **h** Representative images of IF staining of CTSD (green), LAMP1 (red), and p62 (white) in the IPL of post-mortem human brain tissue. Scale bar, 100 μm. **i** Quantification of the mean intensity of (Left) CTSD, (Middle) LAMP1, and (Right) p62 across different disease conditions (*P < 0.05; unpaired *t*-test, *n* = 3 cases/group). **j** Left Panel: Representative images of IF staining of CTSD (green) and LAMP1 (red) in AAV-CT and AAV-BAG3-injected mice in the CA2–CA3 region of the hippocampus. Scale bar, 200 μm. Right Panel: Quantification of the mean intensity of CTSD and LAMP1 in AAV-CT vs AAV-BAG3-injected mice (*P < 0.05 and **P < 0.01; Mann–Whitney test, *n* = 3–5 mice/group, 3 image/mouse). **k** Left Panel: Representative images of IF staining of p62 (green) in the CA1 region of the hippocampus in AAV-CT and AAV-BAG3-injected mice. Scale bar, 50 μm (low-magnification images), 10 μm (inset images). Right Panel: Quantification of the number of cells with aggregated p62 puncta in AAV-CT and AAV-BAG3-injected mice (*P < 0.05; Mann–Whitney test, *n* = 3–5 mice/group, 3 image/mouse). Nuclei for IF images were counterstained by Hoechst 33342

Since BAG3, a prospective modulator of the autophagic flux, was reduced in neurons and OLG with ptau accumulation, global dysfunction of the ALP may exist after TBI in mouse models and in the human condition. To better understand autophagy dynamics after TBI, we measured the immunoreactivity of CTSD (Cathepsin D, a lysosomal protease) [39], LAMP1 (Lysosomal-associated membrane protein 1, a regulator of lysosomal integrity, pH and catabolism) [2, 20, 115], and p62 (an autophagic substrate) [82] in mouse models of TBI and post-mortem human brain tissue with TBI history and AD. After TBI in WT and hTKI mice, levels of CTSD and LAMP1 in the CA1 region of the hippocampus were significantly reduced compared to the sham group, respectively (Fig. 8a–d). Furthermore, there was an increased number of cells with aggregated p62 puncta in the RSC of both mouse models (Fig. 8e–g). Thus, these data show that TBI induces dysfunction of the ALP, which in turn may increase the quantity of ptau (Fig. 1b–e), leading to cognitive dysfunction (Fig. 2e–h). Again, we desired to investigate these results in the post-mortem human brain tissue from cases with TBI, ADwTBI, and AD. In accordance with the mouse data, our panel of ALP-associated markers (CTSD, LAMP1, and p62) shows significant alterations in the post-mortem cases compared to the CT (Fig. 8h, i), suggesting that autophagy and lysosome degradation system are significantly perturbed following TBI and in AD. These results suggest that disruption of the ALP after TBI inhibits clearance of protein aggregates, leading to their accumulation and cellular stress as observed in several neurodegenerative diseases. [3, 10, 67, 86, 105].

Additionally, to assess BAG3 as a therapeutic strategy for the clearance of protein aggregates, it is imperative to understand how overexpression of BAG3 impacts autophagy dynamics in vivo*.* Overexpression of neuronal BAG3 prior to TBI in hTKI mice (AAV-BAG3) resulted in a significant increase in CTSD and LAMP1 (Fig. 8j), while reducing the number of p62 puncta (Fig. 8k) compared to the controls. Thus, BAG3 overexpression is sufficient to increase ALP efficiency in vivo*.* Our results suggest that TBI results in a reduction of BAG3 and ALP inefficiencies, which lead to ptau accumulation and memory impairments in mouse models and post-mortem human brain tissue. Importantly, BAG3 overexpression attenuates TBI-related ALP deficiencies, and reduces global ptau, while improving cognitive function, suggesting that therapies targeting BAG3 may help regulate ALP inadequacies after the injury.

## Discussion

BAG3 has been found to play an important role in promoting the degradation and clearance of pathological protein aggregates, such as tau and alpha-synuclein [22, 42, 49, 52–54, 90]. However, its cell-type-specific role in TBI-induced pathology and cognitive deficits remains unknown. In this study, we found that a single unilateral TBI altered the cell-type-specific expression of BAG3 (Fig. 4 and Supplementary Fig. 6), which is associated with decreased ALP-associated genes and increased p62 puncta formation (Fig. 8a–g), ptau in EX neurons and OLG (Fig. 1b–e and Supplementary Fig. 2a–d), gliosis (Fig. 1f–k and Supplementary Fig. 3), synaptic dysfunction (Fig. 2a–d), and cognitive deficits (Fig. 2e–h) in WT and hTKI mouse models. Specific overexpression of BAG3 in neurons of hTKI mice attenuated the inhibition of ALP (Fig. 8j, k), ptau (Fig. 6d–f), synaptic dysfunction (Fig. 6g), and cognitive deficits induced by TBI (Fig. 6h–j). Furthermore, knockdown of BAG3 significantly inhibited the autophagic flux, while overexpression of BAG3 significantly increased it in vitro (Fig. 7c–g). In this study, we show a relationship between impairments of the ALP, reduction of BAG3 level, and ptau accumulation in OLG for the first time. Furthermore, while BAG3 has been extensively investigated in its relationship with tau pathology [42, 49, 52–54, 90], we investigated the relationship of BAG3, tau pathology, and cognitive deficits in the context of TBI for the first time. Our findings suggest that targeting neuronal BAG3 and the ALP may be a therapeutic strategy for preventing or reducing tau pathology and cognitive deficits induced by TBI.

The defects of the ALP have been found to be strongly associated with protein aggregates in late-onset neurodegenerative diseases including AD [10, 67]. Promoting the clearance of these aggregates in the brain is typically associated with the improvement of symptoms [110]. Thus, enhancing the ALP activity is an appealing therapeutic intervention [10, 62, 67]. BAG3 (a co-chaperone critical for autophagy) tightly regulates the ALP [85, 89], and has been widely demonstrated to ameliorate tau pathology [10, 17, 61, 62, 67, 109, 110]. Notably, we have previously reported that BAG3 is significantly reduced in neurons of human AD compared to non-dementia controls [22]. Although several studies show increased global levels of BAG3 in AD [6, 46, 90], the observed increase of global BAG3 in the AD condition can be contributable to the significant increase of BAG3 in the astrocytes (Fig. 4a–d and Fig. 5a–c). Thus, investigating the cell type-specific changes of BAG3 in different diseases is of upmost importance to determine the complexity of BAG3 changes that are observed. In this study, we demonstrated that TBI reduced the expression of BAG3 in neurons and OLG (Fig. 4e–h, Supplementary Fig. 6, Fig. 5d–g, and Supplementary Fig. 7) with ptau accumulation, as well as the global reductions in ALP-associated genes, such as CTSD and LAMP1 (Fig. 8a–d, h, i), which have also been altered in AD [58, 60, 88, 98]. Decreased BAG3 correlates well with the reductions in CTSD and LAMP1, and the increases in p62 puncta formation (Fig. 8a–g) and ptau accumulation (Fig. 1b–e and Supplementary Fig. 2a–d, f–i) in EX neurons and OLG in both WT and hTKI mouse models. Although p62 is a well-known marker of autophagic flux [82], investigating the function of the lysosomes is important in understanding the dynamics of ALP. In a rat model of middle cerebral artery occlusion, another model of brain injury, reductions of LAMP1 and CTSD correlated well with autophagy flux disruptions as shown by insoluble p62 accumulation [55]. The decrease of lysosomal markers LAMP1 and CTSD indicated rupture of lysosomal compartments [55]. Thus, the reductions of CTSD and LAMP1, in tandem with p62 accumulation in the mouse models of TBI indicate not only impairment of protein degradation systems, but also lysosomal dysfunction. These dysfunctions in the ALP are recapitulated in human postmortem brains with TBI and/or AD (Fig. 8h, i). Importantly, overexpression of BAG3 in neurons significantly prevented ALP dysfunctions, i.e., enhancing the levels of BAG3, CTSD, and LAMP1 and reducing the levels of p62 puncta and ptau (Fig. 6d–f and Fig. 8j, k). Aside from the direct role of BAG3 in protein sequestration [53], previous investigations suggest that BAG3 expression can have a direct effect on the lysosomes, as shown by increased LAMP2 levels in HT-22 cells [13]. Thus, overexpression of BAG3 prior to TBI likely influences lysosomal integrity and protein sequestration after TBI. Using an autophagy reporter, FUW mCherry-GFP-LC3, we further found knockdown of BAG3 significantly inhibited the autophagy flux, while overexpression of BAG3 significantly increased it (Fig. 7f, g). Analysis of BAG3 interacting proteins revealed that BAG3 binds with Atg3 and Atg7, suggesting that BAG3 may be involved in the maturation of autophagosomes and lysosomal fusion [15]. Thus, the correlation between BAG3 expression and autophagic flux, visualized by the FUW virus, shows the importance of BAG3 in autophagy progression. These findings indicate that BAG3 is involved in the ALP dysfunction and ptau accumulation in EX neurons and OLG induced by TBI.

The accumulation of ptau in EX neurons is consistent with previous reports describing EX neuronal differential vulnerability to ptau accumulation compared to IN neurons [22, 50, 75]. Recent reports have also shown increased ptau in OLG after TBI [32, 116] and in the aging mouse brain [99]. The previous neglect of tau accumulation in OLG may be due to the high enrichment of tau in neurons and the ptau antibodies used to characterize tau hyperphosphorylation. In previous reports and ours, neurons and OLG indeed show ptau labeling in WT control mice [32, 70, 77, 81]. The observed PHF1 + cells in WT sham animals are few in numbers and mostly localized to OLIG2 + cells (Supplementary Fig. 2 and [32]), rather than neurons. Indeed, differing methods of immunohistological staining (i.e., antigen retrieval) or antibodies used to detect ptau may result in the discrepancies regarding ptau accumulation in WT animals and OLG in the literature. OLG are the second cell type with relatively high level of tau although its level is much lower than neurons (https://brainrnaseq.org/). There are two potential reasons for increased ptau in OLG in TBI and AD. One reason is that increased tau expression in OLG enhances the chance of forming more tau pathology since tau is a protein prone to aggregate under pathological conditions. Previous studies have indicated that autophagy flux impairment may exist in oligodendrocytes and oligodendrocyte precursor cells after TBI [86], which may make them susceptible to protein aggregation. The other is pathological tau spreading from neurons to OLG can seed endogenous OLG tau and increase ptau and aggregation in those cells. In this study, we show that TBI can promote ptau in OLG, indicating TBI not only increases the neuronal vulnerability to ptau but also enhances the vulnerability of OLG **(**Fig. 1b–e and Supplementary Fig. 2). The transmission of OLG tau pathology is not affected by neuronal tau ablation [71], suggesting that TBI-mediated tau pathology formation may occur independently in OLG and neurons. However, the relationship between OLG tau and neuronal tau as well as the functional implication of OLG ptau in TBI-induced AD-like pathology and cognitive deficits remains to be studied in future. For example, tau species in OLG in TBI and AD as well as the contribution of OLG tau to OLG dysfunction, demyelination, and neuroinflammation in TBI and AD are unclear.

Interestingly, we also noted that BAG3 protein level is significantly increased in GFAP-positive astrocytes in WT and hTKI mouse models with TBI (Fig. 4a–d) and in human postmortem brains with TBI history (Fig. 5a–c). Although we have not validated if the overexpression of BAG3 specifically in astrocytes can prevent AD-like pathology and cognitive deficits induced by TBI in this study, increased BAG3 in astrocytes is probably a protective and compensatory response of astrocytes to degrade tau in pathological conditions because there is no ptau found in astrocytes (Supplementary Fig. 2g) in animal models with TBI. This is consistent with a recent report showing astrocyte BAG3 expression is itself protective and upregulated in disease-associated astrocytes in human AD [90]. It should be noted that although astrocytes are attempting to clear tau protein aggregates by increasing the expression of BAG3, this compensatory response may not be sufficient to prevent ptau accumulation in neurons or OLG in TBI and AD, perhaps because it occurs too late in the disease course. It is also important to note that astrocytic ptau accumulation has been noted in the hippocampus of human AD [80], in human cases of CTE [1], and in mouse models mimicking the repetitive head injury of CTE [43]. Although in human cases and mouse models, the deposition of ptau occurs mainly in the neurons, whereas astrocytic ptau accumulation is localized specifically at sulci depths [4, 43]. Furthermore, while most mouse models of TBI do not report astrocytic ptau accumulation, one report shows that less than 7% of cells burdened with ptau are astrocytes [43]. Thus, it would be interesting to investigate how BAG3 levels change in the subset of astrocytes afflicted with ptau accumulation in these samples. Future studies targeting BAG3 specifically in astrocytes before TBI or at the early stage of AD are warranted to elucidate the role of astrocyte BAG3 in TBI and AD at the molecular and cellular levels.

Pathological tau accumulation and propagation are usually associated with gliosis, synaptic dysfunction, and memory loss in AD [40, 41, 57]. We indeed found increased dyshomeostatic astrocytes and microglia, which are evidenced by the upregulation of GFAP and IBA-1 immunoreactivity and the decrease in microglia branches (i.e., less area of IBA-1 immunoreactivity per microglia) in both WT and hTKI mouse models with TBI in several regions, both proximal and distal to the injury site (Fig. 1f–k and Supplementary Fig. 3). Dyshomeostatic astrocytes and microglia also increased in regions burdened with tau pathology in human cases of TBI, AD, and AD with TBI history compared to control cases (Supplementary Fig. 5). These results are consistent with many studies that suggest increased reactive gliosis in several brain regions after TBI [94, 102]. Dyshomeostatic microglia and astrocytes can induce alteration in synaptic pruning, resulting in synaptic dysfunction and memory loss in AD [30, 84]. In this study, we also found TBI significantly reduced the immunoreactivity of PSD95 (Fig. 2a–d), but not SNAP-25 (Supplementary Fig. 4a–d), suggesting single TBI mainly affects the postsynaptic functions to induce memory loss. Indeed, previous reports indicate post-synaptic function is impaired at 7 and 30 days post-TBI [66, 100], while presynaptic dysfunction occurs early in the injury cascade 3 days post-injury [24] but recovers at 30 days post-injury [92]. These studies and ours indicate that post-synaptic dysfunctions are disrupted in the chronic phase after TBI injury. Since BAG3 is enriched in the post-synaptic density [117] and BAG3 overexpression has been shown to increase PSD95 in PS19 mice [54], it is possible that TBI-induced post-synaptic disruptions are in part due to BAG3 dyshomeostasis. The relationship between TBI, BAG3, and synaptic integrity warrants greater mechanistic understanding in future. Spatial memory measurements by Y-maze indeed showed that TBI significantly reduced cognitive function, which could be prevented by overexpression of BAG3 specifically in neurons as assessed by both Y-maze and the Morris Water Maze (Fig. 2e–h and Fig. 6h–j). Dyshomeostatic microglia and astrocytes can also affect their abilities to take up, spread, and degrade pathological tau [5, 28, 61, 90]. TBI has been found to promote tau spreading in mouse models [114, 116]. Thus, it will be interesting to investigate the molecular state of microglia, astrocytes, and OLG at different time points after TBI, the neuron–glia communication, and the involvement of glial BAG3 in TBI-induced AD-like pathology and cognitive deficits in future.

Our study has a few limitations that merit future consideration. First, the sample size is not big enough for sex difference analysis although we included both male and female mouse and human samples in this study. Second, although the use of post-mortem human brain tissue can help increase translational relevancy of pre-clinical injury models, we recognize that TBI in the human population is a widely heterogeneous injury, which can make interpretation of the results difficult, especially with small sample sizes. Future studies with large cohorts of post-mortem human TBI tissue may resolve this issue by stratifying samples based on the type and the location of injury suffered, the number of injuries suffered, the duration from injury to death, the severity of the injury, and the age to more quantitatively relate TBI heterogeneity to differing neuropathological features. Third, we chose the CCI model for the TBI because single mild CCI or fluid percussion injury (FPI) did not significantly increase ptau in our models (data not shown). Repetitive mild CCI or FPI, blast, and the closed-head impact model of engineered rotational acceleration (CHIMERA)-TBI models may be used to validate our findings from this study. Fourth, although we have identified BAG3 as a downstream mediator of TBI-induced ALP dysfunction, tau accumulation, gliosis, and cognitive deficits, TBI may alter the expression of many other genes associated with tau protein homeostasis [22] that may contribute to this detrimental phenotype, and the function of these genes in the context of TBI and AD warrants future study. Fifth, although neuronal BAG3 overexpression can attenuate ptau accumulation, deficits in post-synaptic density, and memory deficiencies, it is worth noting that a time course experiment was not done for this study. Thus, treating mice with BAG3 overexpression after TBI merits future investigation to determine whether modulating the ALP is an effective therapy after TBI.

In conclusion, we demonstrate that neuronal BAG3 attenuates tau hyper-phosphorylation and cognitive deficits induced by TBI probably via the increased ALP. Our findings will provide greater insights into the molecular mechanisms underlying the TBI-associated AD and ADRD, and the potential development of novel therapeutics targeting BAG3 for preventing and treating TBI and TBI-associated AD and ADRD.

## Supplementary Information

Below is the link to the electronic supplementary material.

Supplementary file1 (TIF 20473 KB) Supplementary Fig. 1. The working hypothesis of single TBI-mediated AD-like pathology and memory loss. A single TBI results in a reduction in excitatory (EX) neuronal and oligodendrocyte (OLG) BAG3, leading to dysfunction of the autophagy-lysosomal pathway (ALP), as shown by decreased CTSD and LAMP1, and increased p62 puncta formation. Dysfunction of the ALP increases ptau accumulation in EX neurons and OLG, which further leads to synaptic dysfunction (decreased post-synaptic density 95 (PSD95)) and memory loss. Additionally, a single TBI results in gliosis, as shown by increased levels of GFAP in astrocytes and IBA-1 in microglia/macrophages. Notably, astrocytes upregulate BAG3, which may protect them from tau aggregates or facilitate astrocytic tau clearance. The reactive gliosis after TBI may also contribute to synaptic dysfunction and memory loss. Furthermore, intervention by BAG3 overexpression in neurons can attenuate deficiencies in the ALP, decrease ptau, and ameliorate post-synaptic density and memory deficits. The solid lines are based on the findings in this study, while the dotted line is hypothesized bidirectional relationship between gliosis and decreased BAG3 levels in EX neurons and OLG.

Supplementary file2 (TIF 23751 KB) Supplementary Fig. 2. TBI increases tau hyperphosphorylation at different phospho-epitopes and BAG3 overexpression attenuates these changes. a, b, Left panel: Representative IF images of CP13+ pSer202 tau (red) staining co-localized with SATB2+ EX neurons (green) and OLIG2+ OLG (white) (a) in the retrosplenial cortex (RSC) and (b) the hippocampus (HC) region of WT Sham and WT TBI mice. Scale bar, 50 μm (low-magnification images) and 10 μm (inset images). Right Panel: The number of CP13+ cells/μm^2^ was quantitated (a) in the RSC and (b) in the HC of WT Sham and WT TBI mice (*P<0.05; Mann–Whitney test, n=5 mice/group). c, d, Left panel: Representative IF images of CP13+ staining co-localized with SATB2+ EX neurons and OLIG2+ OLG (c) in the RSC and (d) the HC region of hTKI Sham and hTKI TBI. Scale bar, 50 μm (low-magnification images) and 10 μm (inset images). Right Panel: The number of CP13+ cells/μm^2^ was quantitated (c) in the RSC and (d) in the HC of hTKI Sham and hTKI TBI (*P<0.05; Mann–Whitney test, n=6 mice/group). e, Representative IF images of CP13+ (red) and OLIG2 (white) in the CA2–CA3 region of the HC of AAV-CT and AAV-BAG3-injected mice. Scale bar, 50 μm. The number of CP13+ cells/μm^2^ was quantitated in the CA2–CA3 region of HC of AAV-CT and AAV-BAG3-injected mice (Mann–Whitney test, n=4 mice/group). f, Representative IF images of GAD1 (green), PHF1 (red), and SATB2 (white) showing co-localization of PHF1+ ptau with SATB2+ EX neurons but not GAD1+ inhibitory neurons in WT TBI mice. g, Representative IF images of IBA-1 (green), PHF1 (red), and GFAP (white) showing no co-localization between PHF1+ ptau and IBA-1+ microglia/macrophage or GFAP+ astrocytes in WT TBI mice. h, Representative IF images of SATB2 (green), PHF1 (red), and OLIG2 (white) showing co-localization of PHF1+ ptau with the pan-oligodendrocyte marker OLIG2 in WT TBI mice. i, Representative IF images of CC1 (green), S396 (red), and PDGFR-α (white) showing co-localization of S396+ ptau with CC1+ mature oligodendrocytes but not PDGFR-α+ oligodendrocyte precursor cells in WT TBI mice. Scale bar, 10 μm. Nuclei for IF images were counterstained by Hoechst 33342. ns; non-significant.

Supplementary file3 (TIF 33137 KB) Supplementary Fig. 3. TBI increases gliosis in wild-type (WT) and hTKI mice in RSC and LP regions. a, d Representative IF images of GFAP (astrocyte marker) and IBA-1 (microglia/macrophage maker) in the RSC of (a) WT Sham vs WT TBI mice and (d) hTKI Sham vs hTKI TBI mice. Scale bar, 100 μm (low-magnification images) and 20 μm (inset images). b, Left Panel: Quantification of the % of Area of GFAP signal in WT Sham vs WT TBI mice (P=0.0649; Mann–Whitney test, n=6 mice/group). Right Panel: Quantification of the mean intensity of GFAP in WT Sham vs WT TBI mice (*P<0.05; Mann–Whitney test, n=6 mice/group). c, Left Panel: Quantification of the % of Area of IBA-1 in WT Sham vs WT TBI mice (**P<0.01; Mann–Whitney test, n=6 mice/group). Right Panel: Quantification of the mean intensity of IBA-1 in WT Sham vs WT TBI mice (**P<0.01; Mann–Whitney test, n=6 mice/group). e, Left Panel: Quantification of the % of Area of GFAP in hTKI Sham vs hTKI TBI mice (*P<0.05; Mann–Whitney test, n=6 mice/group). Right Panel: Quantification of the mean intensity of GFAP in hTKI Sham vs hTKI TBI mice (*P<0.05; Mann–Whitney test, n=6 mice/group). f, Left Panel: Quantification of the % of Area of IBA-1 in hTKI Sham vs hTKI TBI mice (P=0.3939; ns=non-significant; Mann–Whitney test, n=6 mice/group). Right Panel: Quantification of the average surface area of IBA-1+ cells in hTKI Sham vs hTKI TBI mice (*P<0.05; Mann–Whitney test, n=6 mice/group, average taken from the surface area of 20 individual IBA-1+ cells/mouse). g, Left: Quantification of the mean intensity of GFAP in WT Sham vs WT TBI mice in the lateral posterior nucleus of the thalamus (LP) (**P<0.01; Mann–Whitney test, n=6 mice/group). Right: Quantification of the mean intensity of IBA-1 in WT Sham vs WT TBI mice in the LP (*P<0.05; Mann–Whitney test, n=6 mice/group). h, Left: Quantification of the mean intensity of GFAP in hTKI Sham vs hTKI TBI mice in the LP (**P<0.01; Mann–Whitney test, n=6 mice/group). Right: Quantification of the mean intensity of IBA-1 in hTKI Sham vs hTKI TBI mice in the LP (**P<0.01; Mann–Whitney test, n=6 mice/group). i, Reference mouse brain atlas (https://atlas.brain-map.org/atlas?atlas=602630314) of the coronal mouse brain sections used in this study. Specific regions of interest that were analyzed in this study are annotated (Retrosplenial cortex (RSC: Teal); Corpus Callosum (CC: Black); Hippocampus (HC: Green); Lateral posterior nucleus of the thalamus (LP: Pink). j, Representative IF images of GFAP (green) and IBA-1 (red) to show the cortical damage induced by CCI TBI and the proximal (RSC, CC, HC) and remote locations (LP) analyzed in this study. The sections are annotated in accordance with the regions of interest analyzed in this study. The color labeling scheme is in accordance with the reference brain atlas (See i). RSC: Teal; CC: White; HC: Green; LP: Pink; CCI injury: Yellow. Scale bars, 1 mm. Nuclei for IF images were counterstained by Hoechst 33342. ns; non-significant.

Supplementary file4 (TIF 33615 KB) Supplementary Fig. 4. TBI does not significantly alter the level of pre-synaptic density (SNAP-25) or the exploratory and locomotor activity. a, b, Left Panel: Representative IF images of SNAP-25 (red) immunoreactivity in (a) the RSC and (b) the HC of WT Sham and WT TBI mice. Scale bar, 100 μm. Right Panel: Quantification of the mean intensity of SNAP-25 in (a) the RSC and (b) the HC of WT Sham and WT TBI mice (Mann–Whitney test, n=6 mice/group). c, d, Left Panel: Representative IF images of SNAP-25 (red) immunoreactivity in (c) the RSC and (d) the HC of hTKI Sham and hTKI TBI mice. Scale bar, 100 μm. Right Panel: Quantification of the mean intensity of SNAP-25 in (c) the RSC and (d) the HC of hTKI Sham and hTKI TBI mice (Mann–Whitney test, n=6 mice/group). Nuclei for all images were counterstained by Hoechst 33342. e, Left Panel: Quantification of the total distance traveled in the open-field behavioral test between WT Sham and WT TBI mice. Middle Panel: Quantification of the time spent in the periphery to evaluate the exploratory and locomotor activity in the open-field test between WT Sham and WT TBI mice. Right Panel: Quantification of the number (No.) of rears in the open-field behavior test between WT Sham and WT TBI mice (Mann–Whiney test, n=10 mice/group). f, Left Panel: Quantification of the total distance traveled in the open-field behavioral test between hTKI Sham and hTKI TBI mice. Middle Panel: Quantification of the time spent in the periphery to evaluate the exploratory and locomotor activity in the open-field test between hTKI Sham and hTKI TBI mice. Right Panel: Quantification of the number (No.) of rears in the open-field behavior test between hTKI Sham and hTKI TBI mice (**P<0.01; Mann–Whiney test, n=8 mice/group). ns; non-significant

Supplementary file5 (TIF 31411 KB) Supplementary Fig. 5: TBI and AD increase glial recruitment to neuritic plaques as well as the number of ptau+ cells and neuritic plaques. a, Representative IF images of GFAP (green), IBA-1 (red), and PHF1 (white) showing glial recruitment to neuritic plaque pathology in TBI, ADwTBI, and AD. Scale bar, 50 µm. b, Quantification of the number of GFAP+ cells/mm^2^ surrounding neuritic plaque pathology. Neuritic plaques were defined as extracellular tau aggregates that lacked a central nucleus, as previously described [34] (*P<0.05 TBI vs CT; **P<0.01 ADwTBI vs TBI; ***P<0.001 ADwTBI vs CT, AD vs TBI, AD vs CT; unpaired t-test, n=4-5 cases/group; average of 5 plaques/case with an ROI of 430 µm by 420 µm). c, Quantification of the number of IBA1+ cells/mm^2^ surrounding neuritic plaque pathology (*P<0.05 TBI vs CT, ADwTBI vs TBI, **P<0.01 ADwTBI vs CT, AD vs TBI, AD vs CT; unpaired t-test, n=4-5 cases/group, average of 5 plaques/case with an ROI of 430 µm by 420 µm). d, Correlation between the log10(Number of IBA1+ cells/mm^2^) and the log10(Number of GFAP+ cells/mm^2^) surrounding neuritic plaque pathology (P<0.0001; r=0.8939; Pearson Correlation, n=19). e, Correlation between log10(PHF1 % Area) and log10(Number of GFAP+ cells/mm^2^) surrounding neuritic plaque pathology (P=0.0001; r=0.7692; Pearson Correlation, n=19). f, Correlation between log10(PHF1 % Area) and log10(Number of IBA1+ cells/mm^2^) surrounding neuritic plaque pathology (P<0.0001; r=0.7875; Pearson Correlation, n=19). d, e, and f, blue dots= CT; purple dots= TBI; red dots= ADwTBI; green dots= AD. g, Representative IF images of PHF1 (red) showing a low-magnification image of tau pathology in TBI, ADwTBI, and AD as well as high magnification of neuritic plaques (white boxes) and PHF1+ cells (yellow boxes). Scale bar, 100 µm (high magnification images) and 800 µm (low-magnification images). h, Quantification of the mean intensity of PHF1 in the IPL (*P<0.05 vs CT; Mann–Whitney test, n=4-5 cases/group). i, Quantification of the Number of PHF1+ plaque/mm^2^ in the IPL, where plaques were defined as PHF1 extracellular aggregates that lacked a central nucleus (P=0.052 TBI vs CT; *P<0.05 ADwTBI vs TBI, AD vs TBI; **P<0.01 ADwTBI vs CT, AD vs CT; unpaired t-test, n=4-5 cases/group). j, Quantification of the Number of PHF1+ cells/mm^2^ in the IPL, where PHF1+ cells were defined by PHF1 aggregates that surrounded a central nucleus (*P<0.05 TBI vs CT, AD vs ADwTBI; **P<0.01 ADwTBI vs TBI, AD vs TBI, AD vs CT; ***P<0.001 ADwTBI vs CT; unpaired t-test, n=4-5 cases/group). Nuclei for IF images were counterstained by Hoechst 33342.

Supplementary file6 (TIF 25141 KB) Supplementary Fig. 6. TBI reduces the immunoreactivity of BAG3 in neurons and OLG with ptau accumulation in WT mice. a, c, Representative confocal IF images of BAG3 (green), PHF1 (red), and OLIG2 (white) in the cortex of WT TBI mice. Yellow arrows indicate (a) PHF1+ neurons (PHF1+/OLIG2-) and (c) PHF1+ oligodendrocytes (PHF1+/OLIG2+). White arrows indicate (a) PHF1- neurons (PHF1-/OLIG2-) and (c) PHF1- oligodendrocytes (PHF1-/OLIG2+). Scale bar, 20 μm. b, d, Quantification of the mean intensity of BAG3 in (b) PHF1+ neurons vs PHF1- neurons of WT TBI mice and in (d) PHF1+ oligodendrocytes vs PHF1- oligodendrocytes of WT TBI mice (*P<0.05, ***P<0.001; Mann–Whitney test, n=7 mice/group, average of 10 cells/mouse). e, f, Representative low-magnification images of PHF1 (red), BAG3 (green), and OLIG2 (white) in the cortex of (e) WT TBI and (f) hTKI TBI mice. e, f, (Top) Yellow arrows indicate PHF1+ neurons (PHF1+/OLIG2-) and white arrows indicate PHF1- neurons (PHF1-/OLIG2-) and (Bottom) yellow arrows indicate PHF1+ OLIG (PHF1+/OLIG2+) and white arrows indicate PHF1- OLG (PHF1-/OLIG2+) in (e) WT TBI and (f) hTKI TBI mice. Scale bar, 50 μm. Nuclei for all images were counterstained by Hoechst 33342.

Supplementary file7 (TIF 19408 KB) Supplementary Fig. 7. TBI and AD reduce the immunoreactivity of BAG3 in neurons and OLG with ptau accumulation. a, c, Representative low-magnification IF images of BAG3 (green), PHF1 (red), and OLIG2 (white) in the IPL of postmortem human brain tissue. a, Yellow arrows indicate PHF1+ neurons while white arrows indicate PHF1- neurons across different disease conditions. Scale bar, 100 μm. b, Quantification of the mean intensity of BAG3 in PHF1+ neurons in TBI, ADwTBI, and AD compared to CT neurons (**P<0.01, ***P<0.001 vs CT; Mann–Whitney test, n=4–5 cases/group, average of 10 cells/case). c, Yellow arrows indicate PHF1+ OLG while white arrows indicate PHF1- OLG across different disease conditions. Scale bar, 100 μm. d, Quantification of the mean intensity of BAG3 in PHF1+ OLG in TBI, ADwTBI, and AD compared to CT OLG (****P<0.0001 vs CT; Mann–Whitney test, n=4–5 cases/group, average of 7–10 cells/case).

Supplementary file8 (TIF 32273 KB) Supplementary Fig. 8. TBI increases the global level of BAG3 in WT and hTKI mice and in the human IPL. a, Left Panel: Representative IF images of BAG3 (red) immunoreactivity in the hippocampus (HC) and the corpus callosum (CC) region of WT Sham and WT TBI mice. Scale bar, 500 μm. Right Panel: Quantitation of the mean intensity of BAG3 in the HC and CC of WT Sham and WT TBI mice (**P<0.01; Mann–Whitney test, n=6 mice/group). b, Left Panel: Representative IF images of BAG3 (red) immunoreactivity in the HC and the CC region of hTKI Sham and hTKI TBI mice. Scale bar, 500 μm. Right Panel: Quantitation of the mean intensity of BAG3 in the HC and CC of hTKI Sham and hTKI TBI mice (**P<0.01; Mann–Whitney test, n=6–7 mice/group). c, Left Panel: Representative IF images of BAG3 (red) immunoreactivity in the retrosplenial cortex (RSC) of WT Sham and WT TBI mice. Scale bar, 50 μm. Right Panel: Quantitation of the mean intensity of BAG3 in the RSC of WT Sham and WT TBI mice (Mann–Whitney test, n=7 mice/group). d, Left Panel: Representative IF images of BAG3 (red) immunoreactivity in the RSC of hTKI Sham and hTKI TBI mice. Scale bar, 50 μm. Right Panel: Quantitation of the mean intensity of BAG3 in the RSC of hTKI Sham and hTKI TBI mice (***P<0.001; Mann–Whitney test, n=7 mice/group). e, Left Panel: Representative IF images of BAG3 (green) from the IPL of post-mortem human brain tissue. Scale bar, 200 μm Right Panel: Quantitation of BAG3 mean intensity from CT, TBI, ADwTBI, and AD cases (P=0.0941, **P<0.01, ****P<0.0001 vs CT; Mann–Whitney test, n=4-5 cases per group). Nuclei for all images were counterstained by Hoechst 33342. ns; non-significant.

Supplementary file9 (TIF 21038 KB) Supplementary Fig. 9. Overexpression of BAG3 does not alter gliosis, SNAP-25 level, body weight, or swimming speed of hTKI mice with TBI. a, b, Left Panel: Representative IF images of GFAP (green) and IBA-1 (red) immunoreactivity in the (a) corpus callosum (CC) and (b) the hippocampus (HC) of AAV-CT or AAV-BAG3-injected hTKI mice. Scale bar, 50 μm. Right Panel: Quantitation of the mean intensity of GFAP and IBA-1 in (a) the CC and (b) the HC of AAV-CT and AAV-BAG3-injected mice (Mann–Whitney test, n=4-5 mice/group). c, Left Panel: Representative IF images of SNAP-25 (red) in the HC of AAV-CT and AAV-BAG3-injected mice. Scale bar, 50 μm. Right Panel: Quantitation of the mean intensity of SNAP-25 in the HC of AAV-CT and AAV-BAG3-injected mice (Mann–Whitney test, n=4 mice/group, 2 image/mouse). d, Top: Quantitation of body weight of AAV-CT and AAV-BAG3-injected mice before Morris Water Maze (MWM) behavioral test. Middle: Quantitation of mean swimming velocity (m/s) of AAV-CT and AAV-BAG3-injected mice during the 2-hr MWM trial. Bottom: Quantitation of mean swimming velocity (m/s) of AAV-CT and AAV-BAG3 mice during the 24-hr MWM trial (Mann–Whitney test, n=7 mice/group). Nuclei for all images were counterstained by Hoechst 33342. ns; non-significant.

Supplementary file10 (TIF 30625 KB) Supplementary Fig. 10. Full-length Western Blot images of protein lysates from human brains, AAV9-injected mouse brains, and HEK293 cells. a, b, Original WB images of Fig. 3e from the inferior parietal lobe of post-mortem human brain tissue lysate from Control (CT), Traumatic Brain Injury (TBI), AD with a history of TBI (ADwTBI), and Alzheimer’s Disease (AD). a, Left Panel: Mouse anti-PHF1 at low exposure. Right Panel: Mouse anti-PHF1 at high exposure. b, Left Panel: Rabbit anti-TauC. Right Panel: Rabbit anti-GAPDH. c, Original WB images of Fig. 6c, e, f from hippocampal and cortex lysate of hTKI AAV-CT or hTKI AAV-BAG3 TBI mice. Left Panel: WB probed with rabbit anti-BAG3 and mouse anti-GAPDH (loading control). Middle Panel: WB probed with mouse anti-PHF1 and rabbit anti-GAPDH. Right Panel: WB probed with rabbit anti-TauC and mouse anti-GAPDH. d, Original WB images of Fig. 7c from HEK293 cell lysate after treatment with lentiviruses to knock-down or overexpress BAG3. Top: WB probed with rabbit anti-BAG3. Bottom Left: WB probed with mouse anti-SQSTM1 (p62). Bottom Right: WB probed with rabbit anti-GAPDH.

## Data Availability

The data used to generate the results that support the findings are all included in the manuscript. Source data can be requested from the corresponding author upon reasonable request.
