# Supplementary figures and images for: Neuronal BAG3 attenuates tau hyperphosphorylation, synaptic dysfunction, and cognitive deficits induced by traumatic brain injury via the regulation of autophagy-lysosome pathway
Source: Acta Neuropathol. 2024 Oct 11;148(1):52. doi: 10.1007/s00401-024-02810-1 (PMC11469979; doi:10.1007/s00401-024-02810-1)

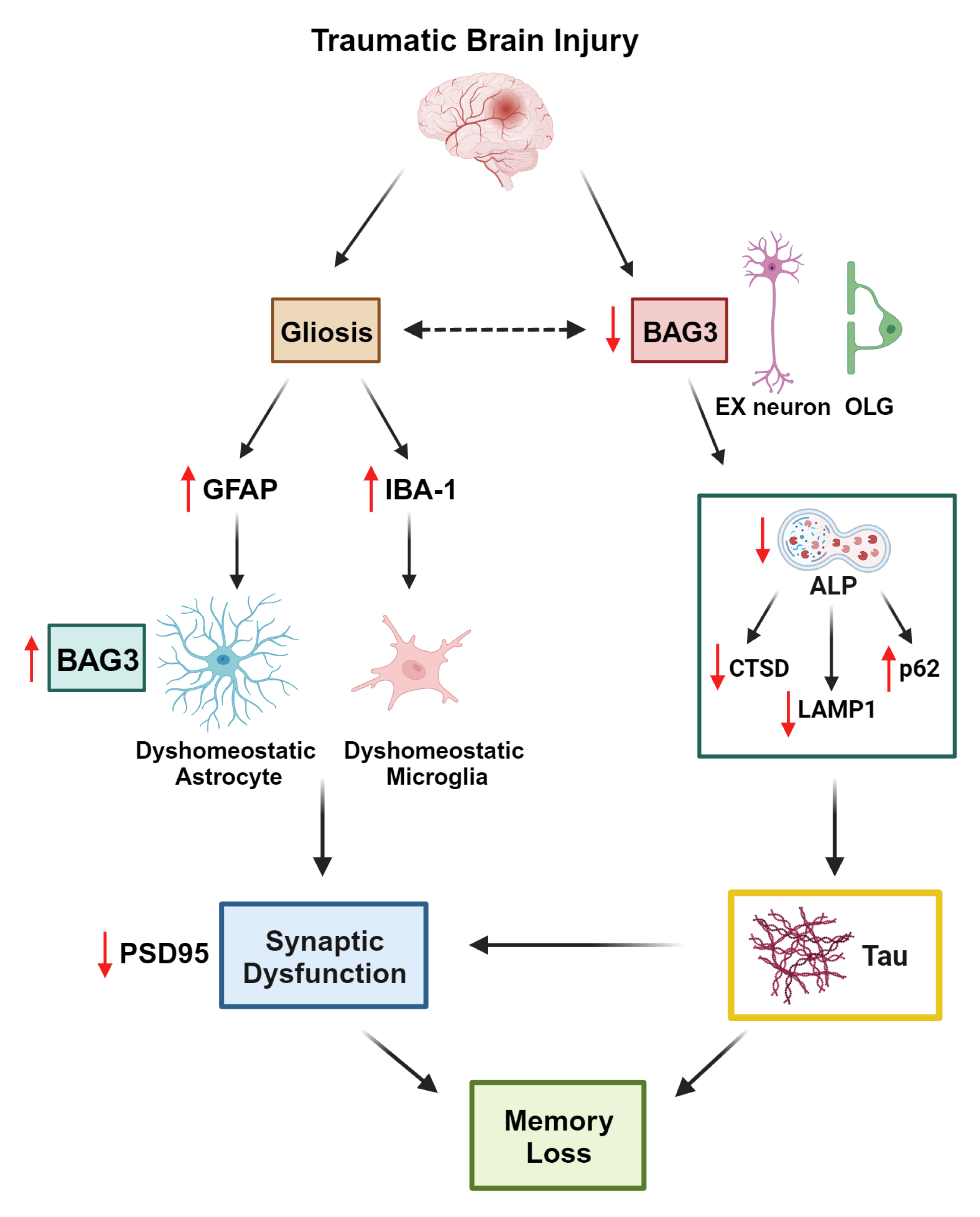

Supplement: Supplementary file 1 — Supplementary file1 (TIF 20473 KB) Supplementary Fig. 1. The working hypothesis of single TBI-mediated AD-like pathology and memory loss. A single TBI results in a reduction in excitatory (EX) neuronal and oligodendrocyte (OLG) BAG3, leading to dysfunction of the autophagy-lysosomal pathway (ALP), as shown by decreased CTSD and LAMP1, and increased p62 puncta formation. Dysfunction of the ALP increases ptau accumulation in EX neurons and OLG, which further leads to synaptic dysfunction (decreased post-synaptic density 95 (PSD95)) and memory loss. Additionally, a single TBI results in gliosis, as shown by increased levels of GFAP in astrocytes and IBA-1 in microglia/macrophages. Notably, astrocytes upregulate BAG3, which may protect them from tau aggregates or facilitate astrocytic tau clearance. The reactive gliosis after TBI may also contribute to synaptic dysfunction and memory loss. Furthermore, intervention by BAG3 overexpression in neurons can attenuate deficiencies in the ALP, decrease ptau, and ameliorate post-synaptic density and memory deficits. The solid lines are based on the findings in this study, while the dotted line is hypothesized bidirectional relationship between gliosis and decreased BAG3 levels in EX neurons and OLG. [file 401_2024_2810_MOESM1_ESM.tif]

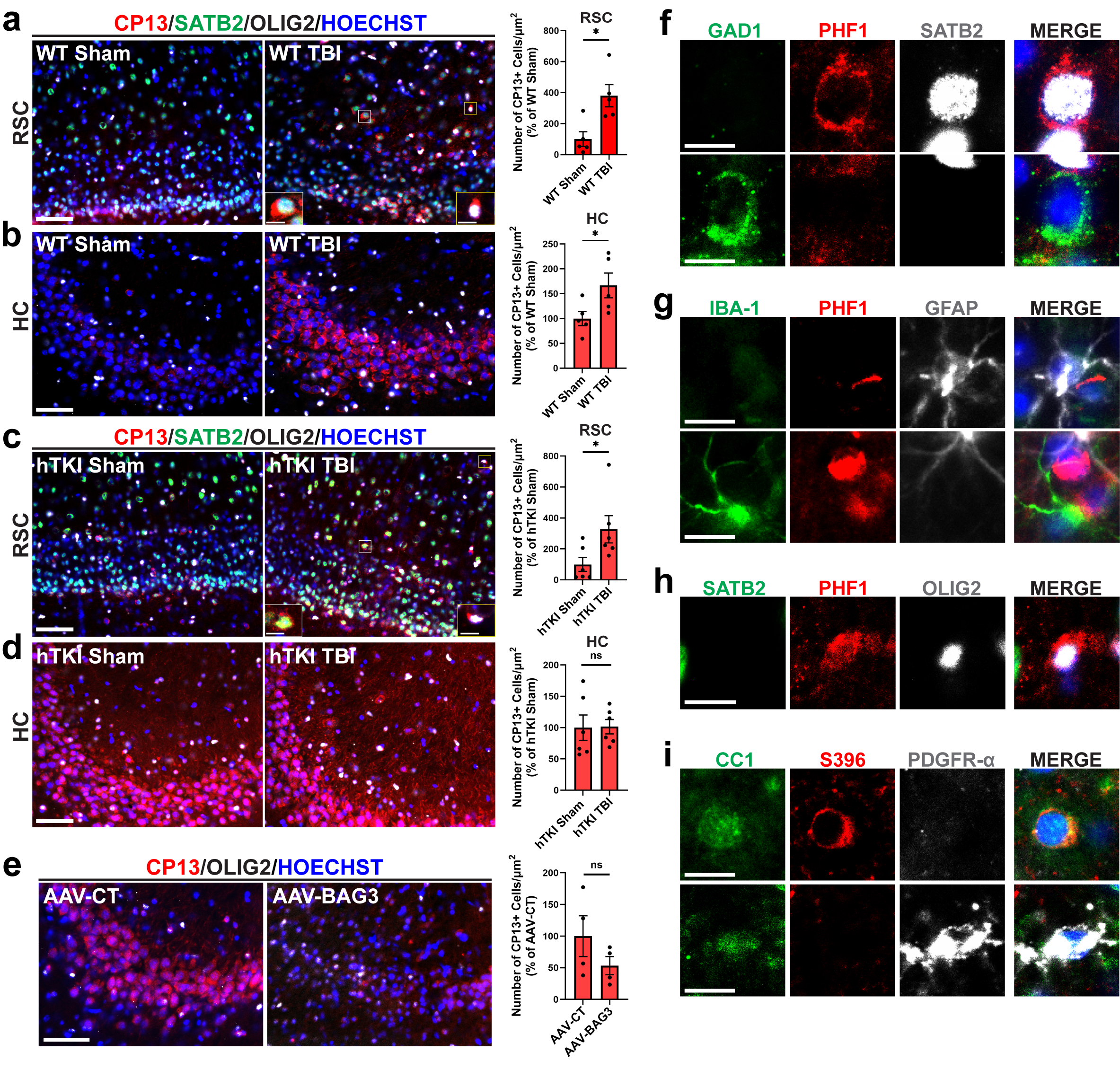

Supplement: Supplementary file 2 — Supplementary file2 (TIF 23751 KB) Supplementary Fig. 2. TBI increases tau hyperphosphorylation at different phospho-epitopes and BAG3 overexpression attenuates these changes. a, b, Left panel: Representative IF images of CP13+ pSer202 tau (red) staining co-localized with SATB2+ EX neurons (green) and OLIG2+ OLG (white) (a) in the retrosplenial cortex (RSC) and (b) the hippocampus (HC) region of WT Sham and WT TBI mice. Scale bar, 50 μm (low-magnification images) and 10 μm (inset images). Right Panel: The number of CP13+ cells/μm2 was quantitated (a) in the RSC and (b) in the HC of WT Sham and WT TBI mice (*P<0.05; Mann–Whitney test, n=5 mice/group). c, d, Left panel: Representative IF images of CP13+ staining co-localized with SATB2+ EX neurons and OLIG2+ OLG (c) in the RSC and (d) the HC region of hTKI Sham and hTKI TBI. Scale bar, 50 μm (low-magnification images) and 10 μm (inset images). Right Panel: The number of CP13+ cells/μm2 was quantitated (c) in the RSC and (d) in the HC of hTKI Sham and hTKI TBI (*P<0.05; Mann–Whitney test, n=6 mice/group). e, Representative IF images of CP13+ (red) and OLIG2 (white) in the CA2–CA3 region of the HC of AAV-CT and AAV-BAG3-injected mice. Scale bar, 50 μm. The number of CP13+ cells/μm2 was quantitated in the CA2–CA3 region of HC of AAV-CT and AAV-BAG3-injected mice (Mann–Whitney test, n=4 mice/group). f, Representative IF images of GAD1 (green), PHF1 (red), and SATB2 (white) showing co-localization of PHF1+ ptau with SATB2+ EX neurons but not GAD1+ inhibitory neurons in WT TBI mice. g, Representative IF images of IBA-1 (green), PHF1 (red), and GFAP (white) showing no co-localization between PHF1+ ptau and IBA-1+ microglia/macrophage or GFAP+ astrocytes in WT TBI mice. h, Representative IF images of SATB2 (green), PHF1 (red), and OLIG2 (white) showing co-localization of PHF1+ ptau with the pan-oligodendrocyte marker OLIG2 in WT TBI mice. i, Representative IF images of CC1 (green), S396 (red), and PDGFR-α (white) showing co-l [file 401_2024_2810_MOESM2_ESM.tif]

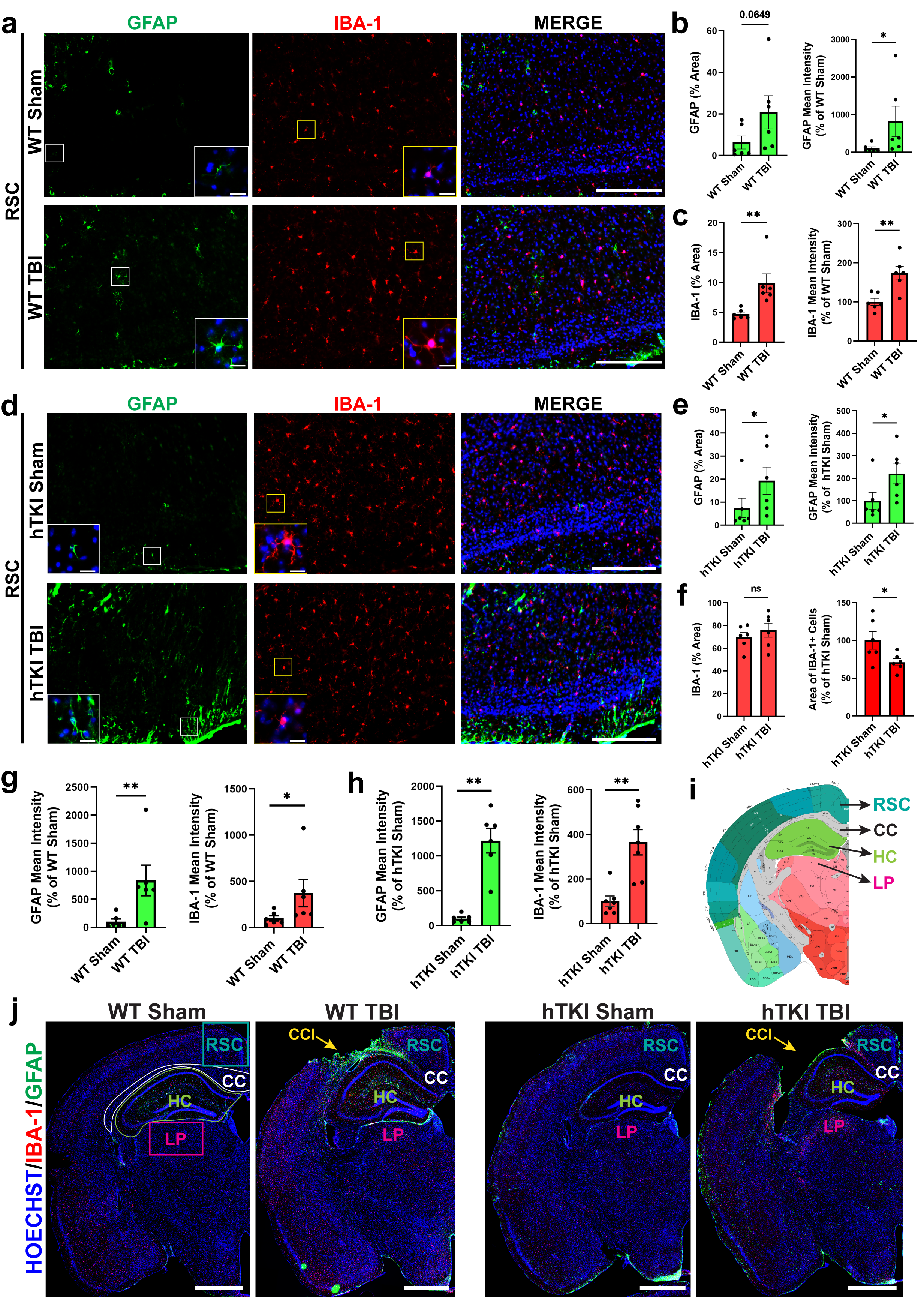

Supplement: Supplementary file 3 — Supplementary file3 (TIF 33137 KB) Supplementary Fig. 3. TBI increases gliosis in wild-type (WT) and hTKI mice in RSC and LP regions. a, d Representative IF images of GFAP (astrocyte marker) and IBA-1 (microglia/macrophage maker) in the RSC of (a) WT Sham vs WT TBI mice and (d) hTKI Sham vs hTKI TBI mice. Scale bar, 100 μm (low-magnification images) and 20 μm (inset images). b, Left Panel: Quantification of the % of Area of GFAP signal in WT Sham vs WT TBI mice (P=0.0649; Mann–Whitney test, n=6 mice/group). Right Panel: Quantification of the mean intensity of GFAP in WT Sham vs WT TBI mice (*P<0.05; Mann–Whitney test, n=6 mice/group). c, Left Panel: Quantification of the % of Area of IBA-1 in WT Sham vs WT TBI mice (**P<0.01; Mann–Whitney test, n=6 mice/group). Right Panel: Quantification of the mean intensity of IBA-1 in WT Sham vs WT TBI mice (**P<0.01; Mann–Whitney test, n=6 mice/group). e, Left Panel: Quantification of the % of Area of GFAP in hTKI Sham vs hTKI TBI mice (*P<0.05; Mann–Whitney test, n=6 mice/group). Right Panel: Quantification of the mean intensity of GFAP in hTKI Sham vs hTKI TBI mice (*P<0.05; Mann–Whitney test, n=6 mice/group). f, Left Panel: Quantification of the % of Area of IBA-1 in hTKI Sham vs hTKI TBI mice (P=0.3939; ns=non-significant; Mann–Whitney test, n=6 mice/group). Right Panel: Quantification of the average surface area of IBA-1+ cells in hTKI Sham vs hTKI TBI mice (*P<0.05; Mann–Whitney test, n=6 mice/group, average taken from the surface area of 20 individual IBA-1+ cells/mouse). g, Left: Quantification of the mean intensity of GFAP in WT Sham vs WT TBI mice in the lateral posterior nucleus of the thalamus (LP) (**P<0.01; Mann–Whitney test, n=6 mice/group). Right: Quantification of the mean intensity of IBA-1 in WT Sham vs WT TBI mice in the LP (*P<0.05; Mann–Whitney test, n=6 mice/group). h, Left: Quantification of the mean intensity of GFAP in hTKI Sham vs hTKI TBI mice in the LP (**P<0.01; Mann–Whitney test, n=6 mice/group). [file 401_2024_2810_MOESM3_ESM.tif]

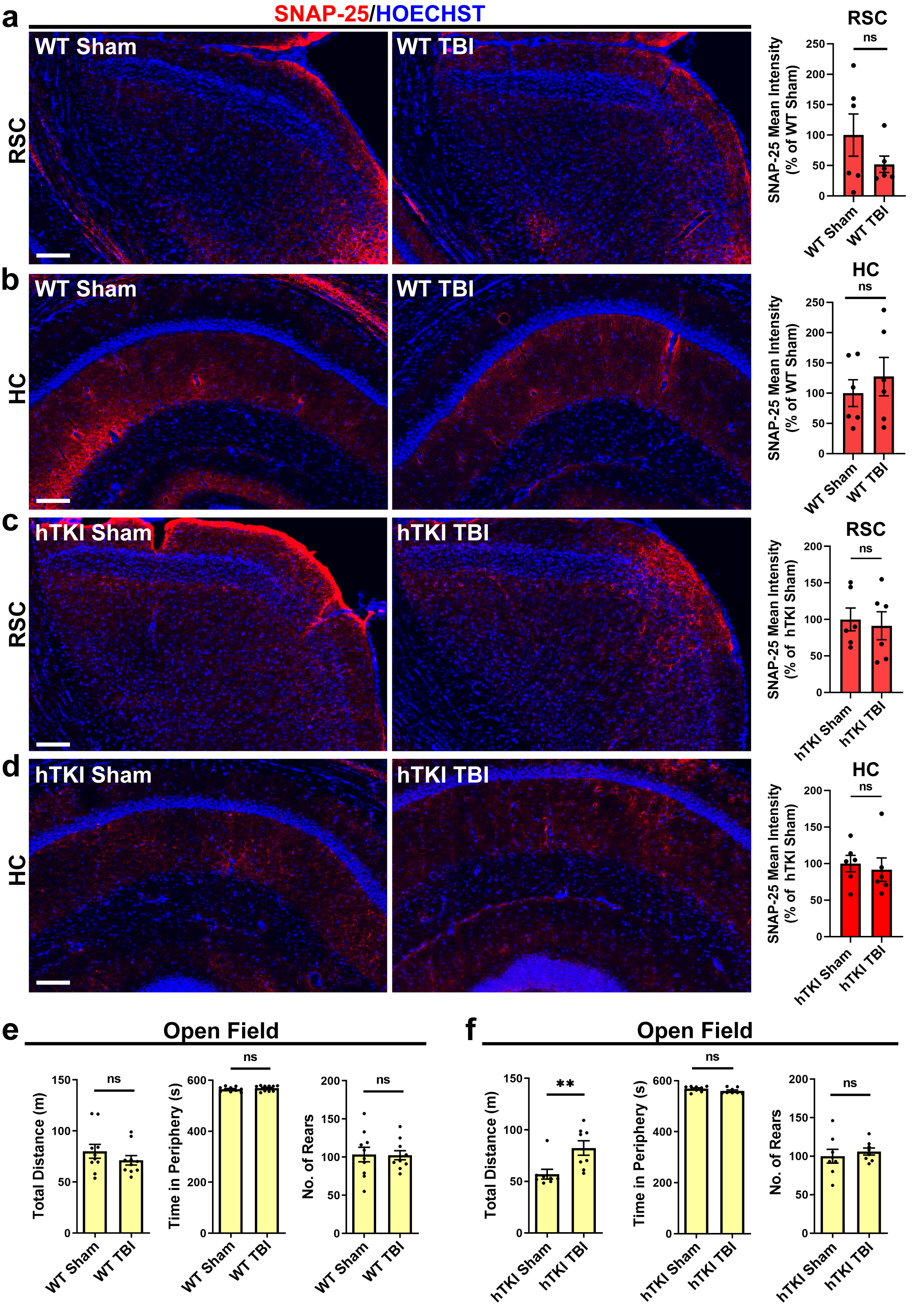

Supplement: Supplementary file 4 — Supplementary file4 (TIF 33615 KB) Supplementary Fig. 4. TBI does not significantly alter the level of pre-synaptic density (SNAP-25) or the exploratory and locomotor activity. a, b, Left Panel: Representative IF images of SNAP-25 (red) immunoreactivity in (a) the RSC and (b) the HC of WT Sham and WT TBI mice. Scale bar, 100 μm. Right Panel: Quantification of the mean intensity of SNAP-25 in (a) the RSC and (b) the HC of WT Sham and WT TBI mice (Mann–Whitney test, n=6 mice/group). c, d, Left Panel: Representative IF images of SNAP-25 (red) immunoreactivity in (c) the RSC and (d) the HC of hTKI Sham and hTKI TBI mice. Scale bar, 100 μm. Right Panel: Quantification of the mean intensity of SNAP-25 in (c) the RSC and (d) the HC of hTKI Sham and hTKI TBI mice (Mann–Whitney test, n=6 mice/group). Nuclei for all images were counterstained by Hoechst 33342. e, Left Panel: Quantification of the total distance traveled in the open-field behavioral test between WT Sham and WT TBI mice. Middle Panel: Quantification of the time spent in the periphery to evaluate the exploratory and locomotor activity in the open-field test between WT Sham and WT TBI mice. Right Panel: Quantification of the number (No.) of rears in the open-field behavior test between WT Sham and WT TBI mice (Mann–Whiney test, n=10 mice/group). f, Left Panel: Quantification of the total distance traveled in the open-field behavioral test between hTKI Sham and hTKI TBI mice. Middle Panel: Quantification of the time spent in the periphery to evaluate the exploratory and locomotor activity in the open-field test between hTKI Sham and hTKI TBI mice. Right Panel: Quantification of the number (No.) of rears in the open-field behavior test between hTKI Sham and hTKI TBI mice (**P<0.01; Mann–Whiney test, n=8 mice/group). ns; non-significant [file 401_2024_2810_MOESM4_ESM.tif]

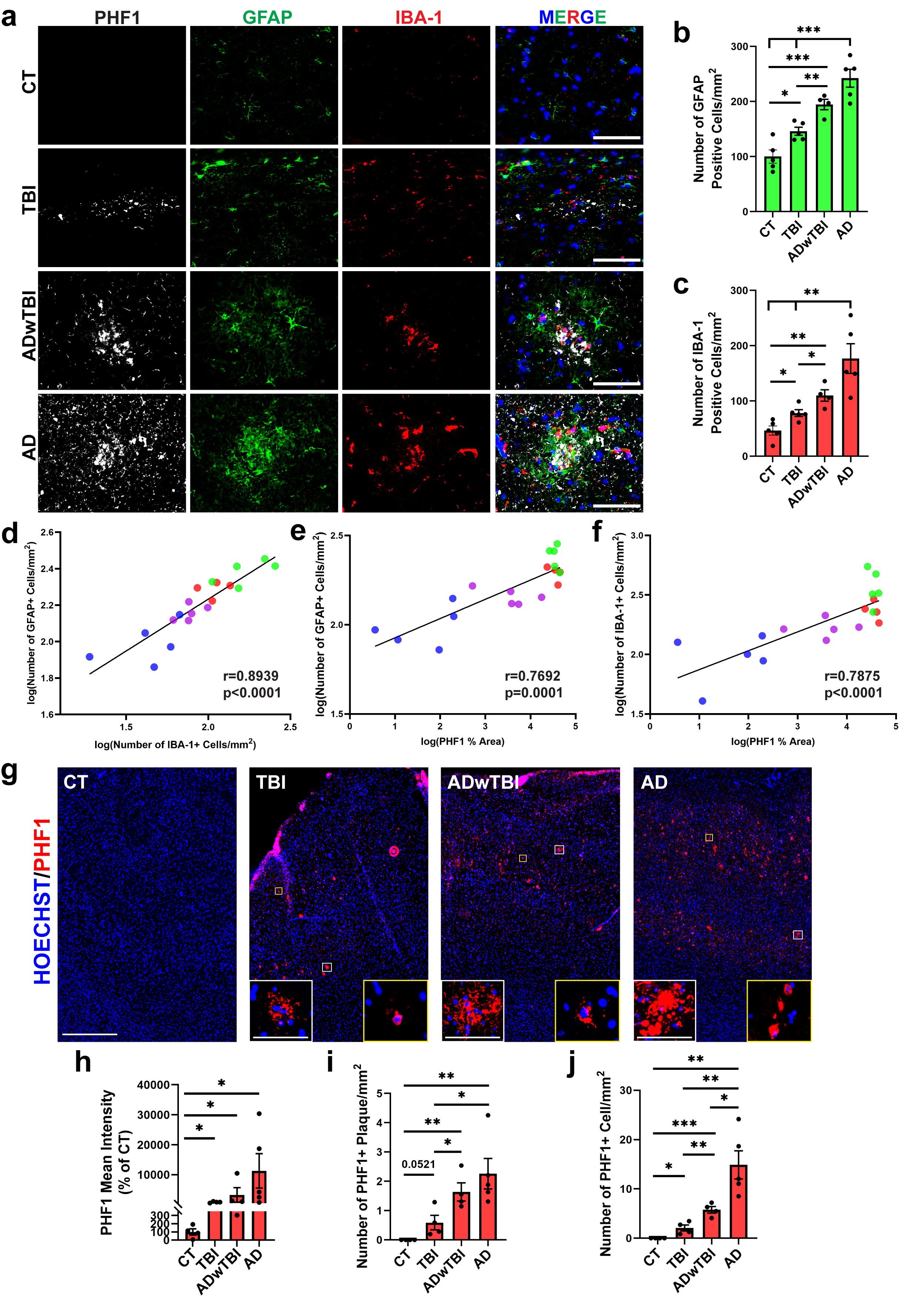

Supplement: Supplementary file 5 — Supplementary file5 (TIF 31411 KB) Supplementary Fig. 5: TBI and AD increase glial recruitment to neuritic plaques as well as the number of ptau+ cells and neuritic plaques. a, Representative IF images of GFAP (green), IBA-1 (red), and PHF1 (white) showing glial recruitment to neuritic plaque pathology in TBI, ADwTBI, and AD. Scale bar, 50 µm. b, Quantification of the number of GFAP+ cells/mm2 surrounding neuritic plaque pathology. Neuritic plaques were defined as extracellular tau aggregates that lacked a central nucleus, as previously described [34] (*P<0.05 TBI vs CT; **P<0.01 ADwTBI vs TBI; ***P<0.001 ADwTBI vs CT, AD vs TBI, AD vs CT; unpaired t-test, n=4-5 cases/group; average of 5 plaques/case with an ROI of 430 µm by 420 µm). c, Quantification of the number of IBA1+ cells/mm2 surrounding neuritic plaque pathology (*P<0.05 TBI vs CT, ADwTBI vs TBI, **P<0.01 ADwTBI vs CT, AD vs TBI, AD vs CT; unpaired t-test, n=4-5 cases/group, average of 5 plaques/case with an ROI of 430 µm by 420 µm). d, Correlation between the log10(Number of IBA1+ cells/mm2) and the log10(Number of GFAP+ cells/mm2) surrounding neuritic plaque pathology (P<0.0001; r=0.8939; Pearson Correlation, n=19). e, Correlation between log10(PHF1 % Area) and log10(Number of GFAP+ cells/mm2) surrounding neuritic plaque pathology (P=0.0001; r=0.7692; Pearson Correlation, n=19). f, Correlation between log10(PHF1 % Area) and log10(Number of IBA1+ cells/mm2) surrounding neuritic plaque pathology (P<0.0001; r=0.7875; Pearson Correlation, n=19). d, e, and f, blue dots= CT; purple dots= TBI; red dots= ADwTBI; green dots= AD. g, Representative IF images of PHF1 (red) showing a low-magnification image of tau pathology in TBI, ADwTBI, and AD as well as high magnification of neuritic plaques (white boxes) and PHF1+ cells (yellow boxes). Scale bar, 100 µm (high magnification images) and 800 µm (low-magnification images). h, Quantification of the mean intensity of PHF1 in the IPL (*P<0.05 vs CT; Mann–Whitney test, n [file 401_2024_2810_MOESM5_ESM.tif]

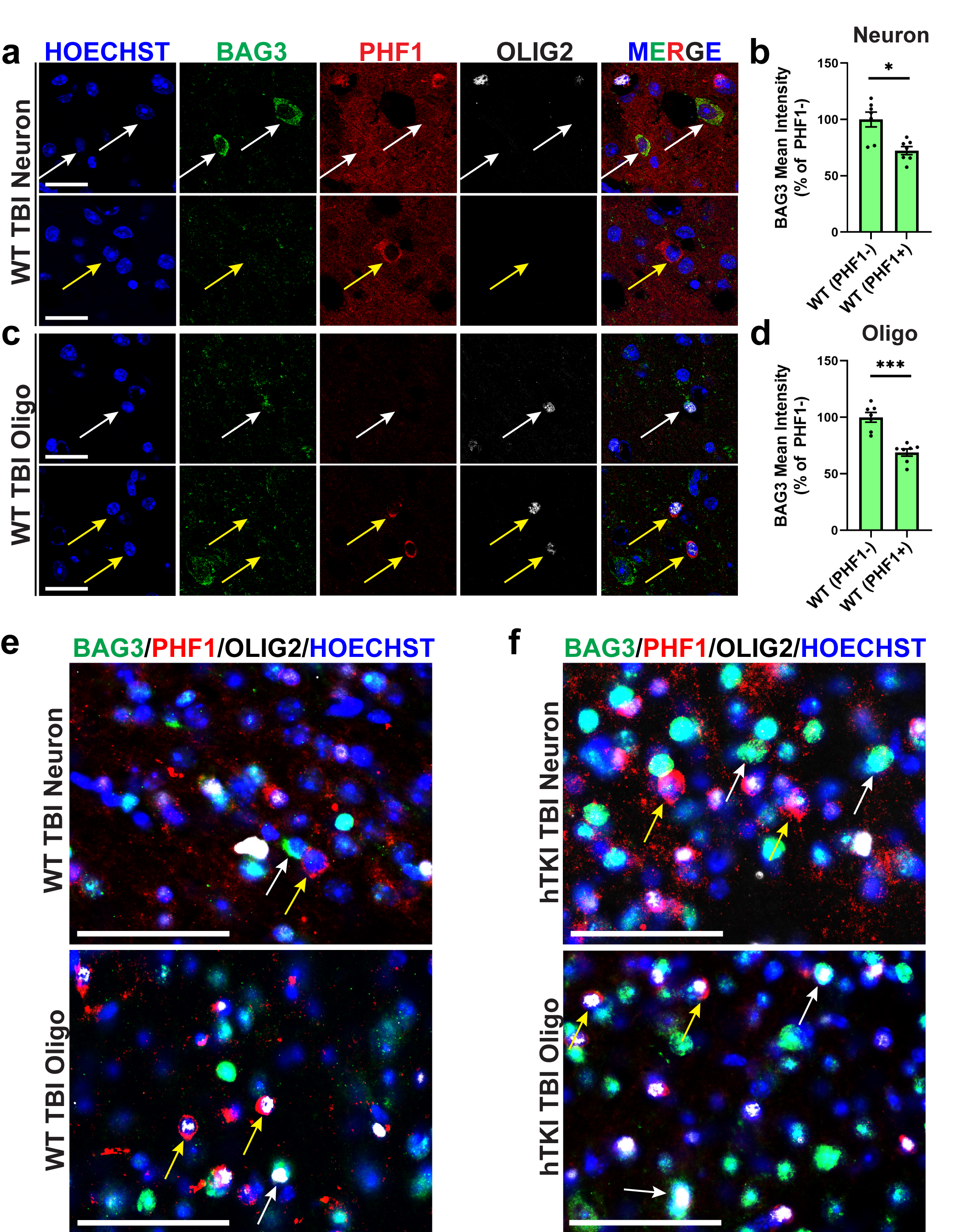

Supplement: Supplementary file 6 — Supplementary file6 (TIF 25141 KB) Supplementary Fig. 6. TBI reduces the immunoreactivity of BAG3 in neurons and OLG with ptau accumulation in WT mice. a, c, Representative confocal IF images of BAG3 (green), PHF1 (red), and OLIG2 (white) in the cortex of WT TBI mice. Yellow arrows indicate (a) PHF1+ neurons (PHF1+/OLIG2-) and (c) PHF1+ oligodendrocytes (PHF1+/OLIG2+). White arrows indicate (a) PHF1- neurons (PHF1-/OLIG2-) and (c) PHF1- oligodendrocytes (PHF1-/OLIG2+). Scale bar, 20 μm. b, d, Quantification of the mean intensity of BAG3 in (b) PHF1+ neurons vs PHF1- neurons of WT TBI mice and in (d) PHF1+ oligodendrocytes vs PHF1- oligodendrocytes of WT TBI mice (*P<0.05, ***P<0.001; Mann–Whitney test, n=7 mice/group, average of 10 cells/mouse). e, f, Representative low-magnification images of PHF1 (red), BAG3 (green), and OLIG2 (white) in the cortex of (e) WT TBI and (f) hTKI TBI mice. e, f, (Top) Yellow arrows indicate PHF1+ neurons (PHF1+/OLIG2-) and white arrows indicate PHF1- neurons (PHF1-/OLIG2-) and (Bottom) yellow arrows indicate PHF1+ OLIG (PHF1+/OLIG2+) and white arrows indicate PHF1- OLG (PHF1-/OLIG2+) in (e) WT TBI and (f) hTKI TBI mice. Scale bar, 50 μm. Nuclei for all images were counterstained by Hoechst 33342. [file 401_2024_2810_MOESM6_ESM.tif]

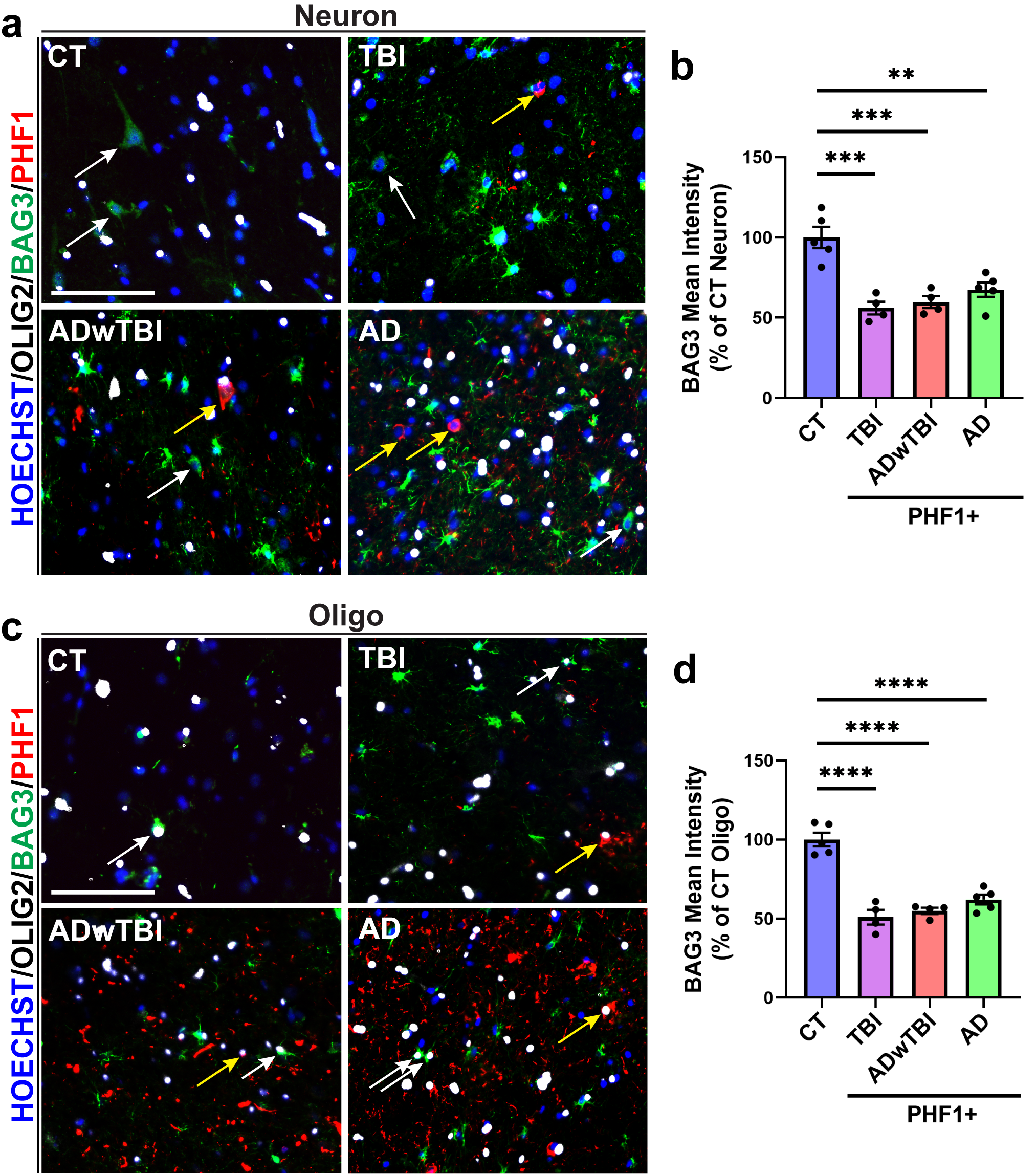

Supplement: Supplementary file 7 — Supplementary file7 (TIF 19408 KB) Supplementary Fig. 7. TBI and AD reduce the immunoreactivity of BAG3 in neurons and OLG with ptau accumulation. a, c, Representative low-magnification IF images of BAG3 (green), PHF1 (red), and OLIG2 (white) in the IPL of postmortem human brain tissue. a, Yellow arrows indicate PHF1+ neurons while white arrows indicate PHF1- neurons across different disease conditions. Scale bar, 100 μm. b, Quantification of the mean intensity of BAG3 in PHF1+ neurons in TBI, ADwTBI, and AD compared to CT neurons (**P<0.01, ***P<0.001 vs CT; Mann–Whitney test, n=4–5 cases/group, average of 10 cells/case). c, Yellow arrows indicate PHF1+ OLG while white arrows indicate PHF1- OLG across different disease conditions. Scale bar, 100 μm. d, Quantification of the mean intensity of BAG3 in PHF1+ OLG in TBI, ADwTBI, and AD compared to CT OLG (****P<0.0001 vs CT; Mann–Whitney test, n=4–5 cases/group, average of 7–10 cells/case). [file 401_2024_2810_MOESM7_ESM.tif]

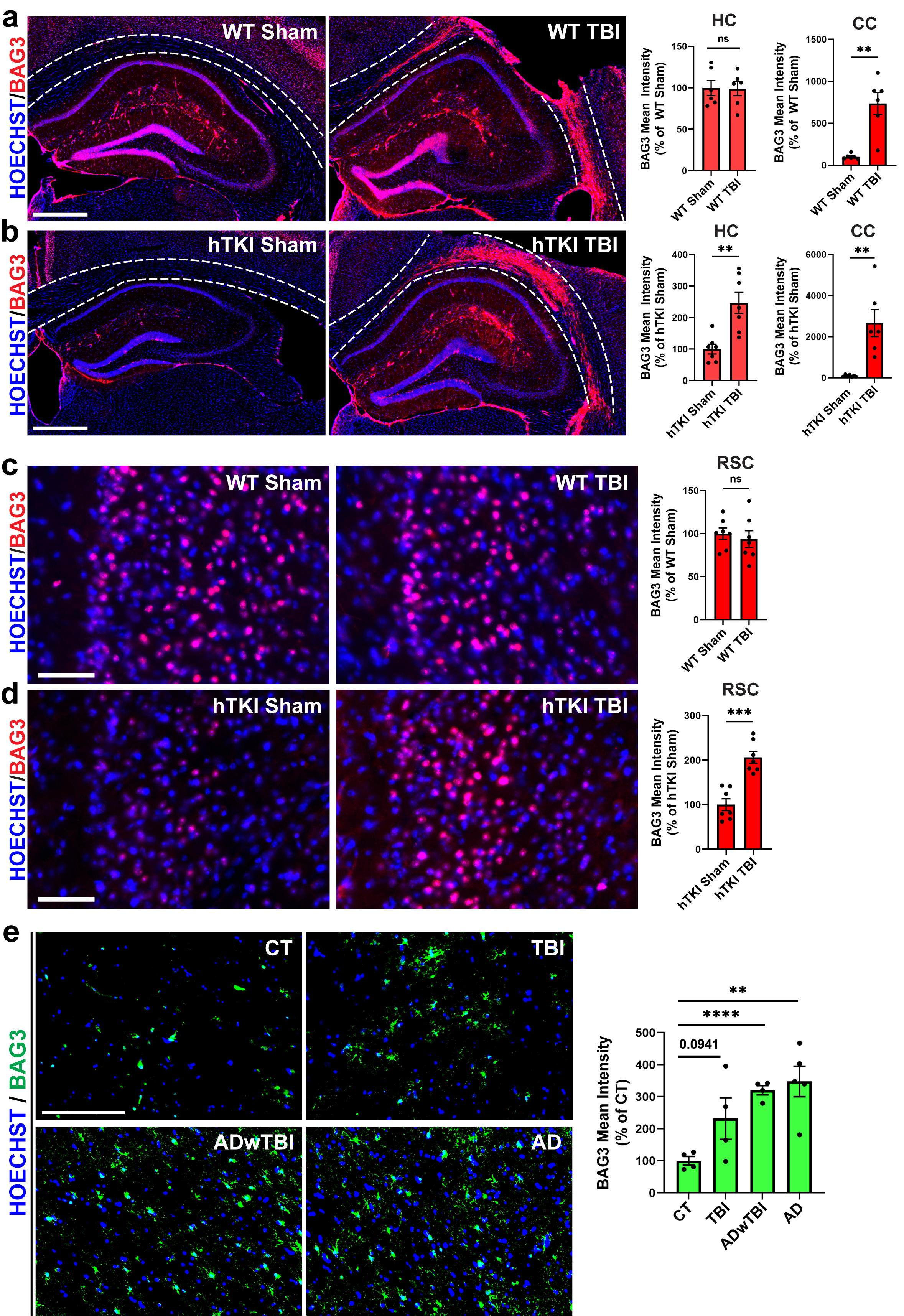

Supplement: Supplementary file 8 — Supplementary file8 (TIF 32273 KB) Supplementary Fig. 8. TBI increases the global level of BAG3 in WT and hTKI mice and in the human IPL. a, Left Panel: Representative IF images of BAG3 (red) immunoreactivity in the hippocampus (HC) and the corpus callosum (CC) region of WT Sham and WT TBI mice. Scale bar, 500 μm. Right Panel: Quantitation of the mean intensity of BAG3 in the HC and CC of WT Sham and WT TBI mice (**P<0.01; Mann–Whitney test, n=6 mice/group). b, Left Panel: Representative IF images of BAG3 (red) immunoreactivity in the HC and the CC region of hTKI Sham and hTKI TBI mice. Scale bar, 500 μm. Right Panel: Quantitation of the mean intensity of BAG3 in the HC and CC of hTKI Sham and hTKI TBI mice (**P<0.01; Mann–Whitney test, n=6–7 mice/group). c, Left Panel: Representative IF images of BAG3 (red) immunoreactivity in the retrosplenial cortex (RSC) of WT Sham and WT TBI mice. Scale bar, 50 μm. Right Panel: Quantitation of the mean intensity of BAG3 in the RSC of WT Sham and WT TBI mice (Mann–Whitney test, n=7 mice/group). d, Left Panel: Representative IF images of BAG3 (red) immunoreactivity in the RSC of hTKI Sham and hTKI TBI mice. Scale bar, 50 μm. Right Panel: Quantitation of the mean intensity of BAG3 in the RSC of hTKI Sham and hTKI TBI mice (***P<0.001; Mann–Whitney test, n=7 mice/group). e, Left Panel: Representative IF images of BAG3 (green) from the IPL of post-mortem human brain tissue. Scale bar, 200 μm Right Panel: Quantitation of BAG3 mean intensity from CT, TBI, ADwTBI, and AD cases (P=0.0941, **P<0.01, ****P<0.0001 vs CT; Mann–Whitney test, n=4-5 cases per group). Nuclei for all images were counterstained by Hoechst 33342. ns; non-significant. [file 401_2024_2810_MOESM8_ESM.tif]

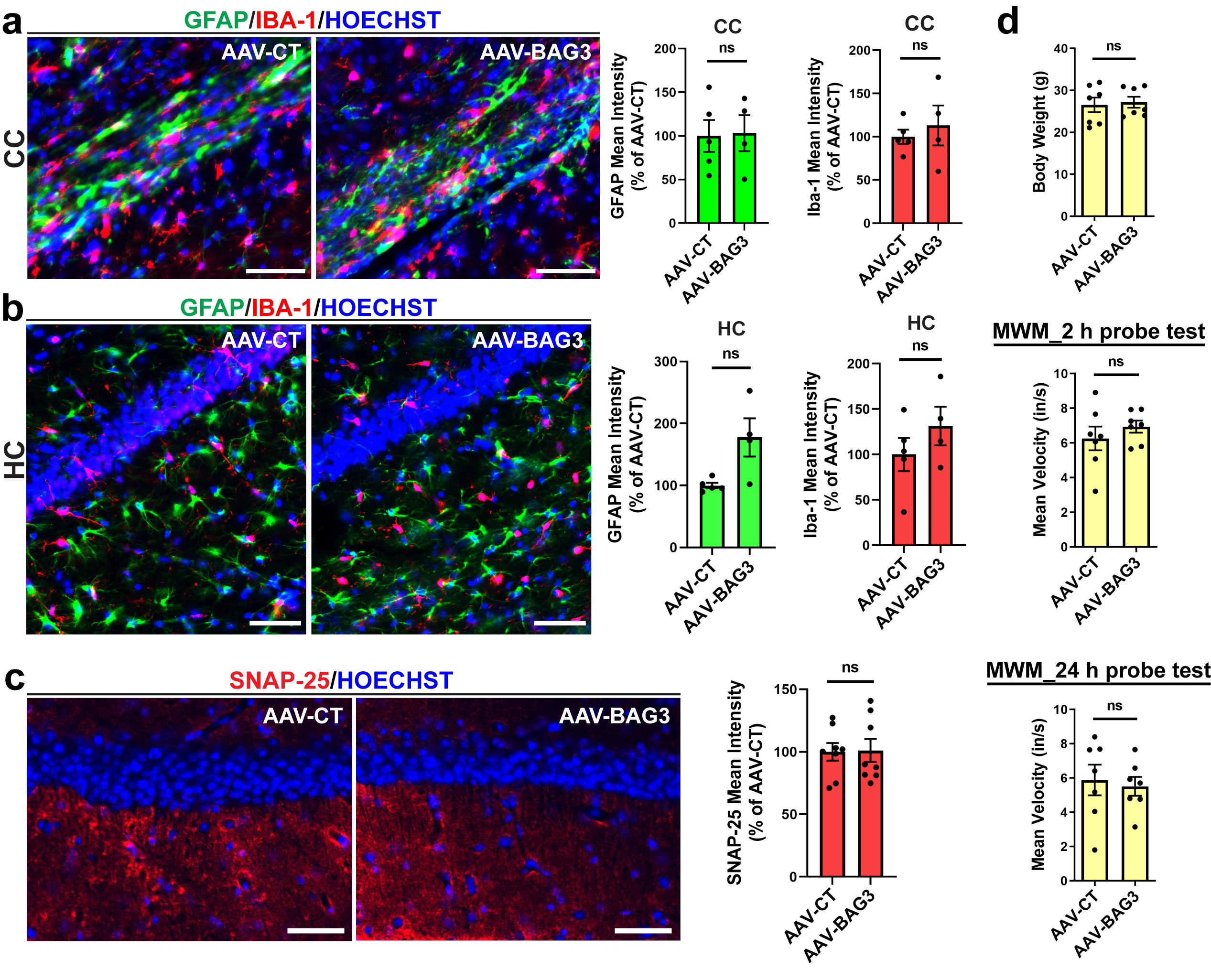

Supplement: Supplementary file 9 — Supplementary file9 (TIF 21038 KB) Supplementary Fig. 9. Overexpression of BAG3 does not alter gliosis, SNAP-25 level, body weight, or swimming speed of hTKI mice with TBI. a, b, Left Panel: Representative IF images of GFAP (green) and IBA-1 (red) immunoreactivity in the (a) corpus callosum (CC) and (b) the hippocampus (HC) of AAV-CT or AAV-BAG3-injected hTKI mice. Scale bar, 50 μm. Right Panel: Quantitation of the mean intensity of GFAP and IBA-1 in (a) the CC and (b) the HC of AAV-CT and AAV-BAG3-injected mice (Mann–Whitney test, n=4-5 mice/group). c, Left Panel: Representative IF images of SNAP-25 (red) in the HC of AAV-CT and AAV-BAG3-injected mice. Scale bar, 50 μm. Right Panel: Quantitation of the mean intensity of SNAP-25 in the HC of AAV-CT and AAV-BAG3-injected mice (Mann–Whitney test, n=4 mice/group, 2 image/mouse). d, Top: Quantitation of body weight of AAV-CT and AAV-BAG3-injected mice before Morris Water Maze (MWM) behavioral test. Middle: Quantitation of mean swimming velocity (m/s) of AAV-CT and AAV-BAG3-injected mice during the 2-hr MWM trial. Bottom: Quantitation of mean swimming velocity (m/s) of AAV-CT and AAV-BAG3 mice during the 24-hr MWM trial (Mann–Whitney test, n=7 mice/group). Nuclei for all images were counterstained by Hoechst 33342. ns; non-significant. [file 401_2024_2810_MOESM9_ESM.tif]

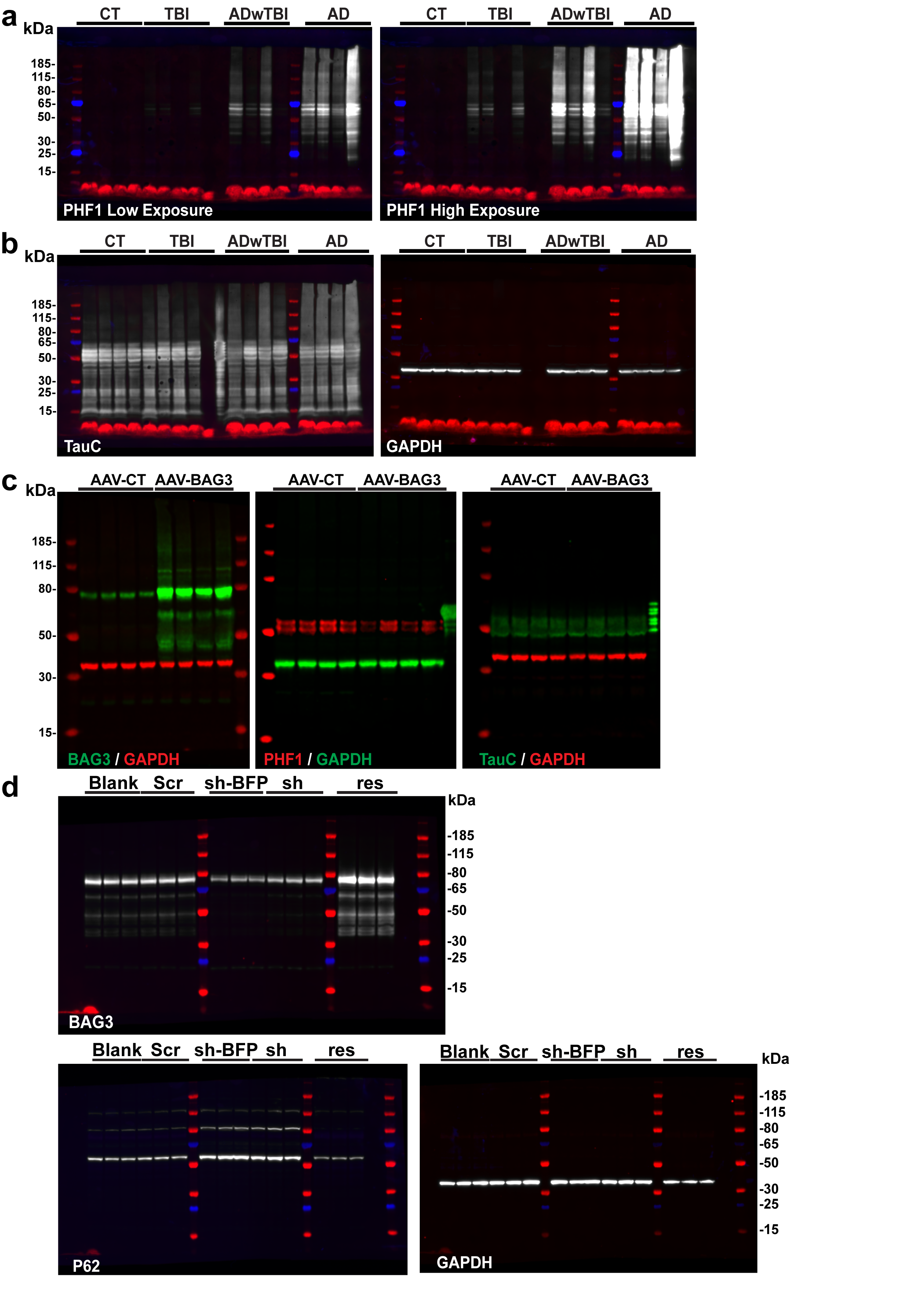

Supplement: Supplementary file 10 — Supplementary file10 (TIF 30625 KB) Supplementary Fig. 10. Full-length Western Blot images of protein lysates from human brains, AAV9-injected mouse brains, and HEK293 cells. a, b, Original WB images of Fig. 3e from the inferior parietal lobe of post-mortem human brain tissue lysate from Control (CT), Traumatic Brain Injury (TBI), AD with a history of TBI (ADwTBI), and Alzheimer’s Disease (AD). a, Left Panel: Mouse anti-PHF1 at low exposure. Right Panel: Mouse anti-PHF1 at high exposure. b, Left Panel: Rabbit anti-TauC. Right Panel: Rabbit anti-GAPDH. c, Original WB images of Fig. 6c, e, f from hippocampal and cortex lysate of hTKI AAV-CT or hTKI AAV-BAG3 TBI mice. Left Panel: WB probed with rabbit anti-BAG3 and mouse anti-GAPDH (loading control). Middle Panel: WB probed with mouse anti-PHF1 and rabbit anti-GAPDH. Right Panel: WB probed with rabbit anti-TauC and mouse anti-GAPDH. d, Original WB images of Fig. 7c from HEK293 cell lysate after treatment with lentiviruses to knock-down or overexpress BAG3. Top: WB probed with rabbit anti-BAG3. Bottom Left: WB probed with mouse anti-SQSTM1 (p62). Bottom Right: WB probed with rabbit anti-GAPDH. [file 401_2024_2810_MOESM10_ESM.tif]
